# Synergy between photonics and biological approaches—A review of combination therapies for cancer theranostics

**DOI:** 10.1063/5.0252574

**Published:** 2025-10-29

**Authors:** Jacob P. Adams, Luke Pauli, Lin Wang, Trisha Valerio, Coline Furrer, Min Li, Naoko Takebe, Joanne Tuohy, Wei R. Chen

**Affiliations:** 1 Stephenson School of Biomedical Engineering, The University of Oklahoma, Norman, Oklahoma 73019, USA; 2 Department of Medicine, The University of Oklahoma Health Sciences Center, Oklahoma City, Oklahoma 73104, USA; 3 Department of Surgery, The University of Oklahoma Health Sciences Center, Oklahoma City, Oklahoma 73104, USA; 4 Stephenson Cancer Center, The University of Oklahoma Health Sciences Center, Oklahoma City, Oklahoma 73104, USA; 5 VA-MD College of Veterinary Medicine, Virginia Tech, Blacksburg, Virginia 24061, USA

## Abstract

Cancer therapies have evolved considerably over the past several decades. Physical interactions, particularly through phototherapy as a new class of treatment modality, have become widely used. Phototherapy utilizes light to combat cancer by generating heat (photothermal therapy), reactive oxygen species (photodynamic therapy), or photochemical internalization, to kill targeted cells. While these therapies have shown promise in pre-clinical studies, they have demonstrated limitations in clinical cancer treatment. Primarily, while phototherapies excel in eliminating primary tumors, they often fail to provide systemic effects, particularly when treating metastatic cancers, in addition to certain undesirable side effects. Phototherapy has been combined with immunotherapy, nanomedicine, and other cancer therapies to overcome specific weaknesses and enhance the therapeutic benefits of individual treatments. These combinations often involve nanomaterials to deliver adjuncts for phototherapy to the tumor site, immune stimulants/adjuvants to enhance the immune response, and immune checkpoint inhibitors to counter immune suppression. Phototherapies may also be combined with specific photonics-related principles to enable simultaneous diagnostic and therapeutic effects, known as theranostics. Herein, we review current approaches to modern cancer therapies, such as nanotechnology-based therapies, immunotherapies, and especially phototherapy, and the combination of these therapies with diagnostic techniques in combating cancer.

## I. INTRODUCTION

Around the world, cancer remains one of the leading causes of mortality, resulting in millions of deaths annually.^1^ While research into cancer treatments has yielded breakthroughs in the fields of surgery, radiation, and chemotherapy, cancer continues to remain a mostly incurable illness. These conventional treatments aim to resect or control tumor growth, but they cannot prevent metastasis, due to immune escape, which accounts for most cancer-related deaths.^2,3^ When tumors evade the immune system, they tend to develop resistance against conventional treatments.^4,5^ Consequently, the side effects of radiation and chemotherapy encouraged research into novel, less toxic, and more effective cancer therapies.

Novel cancer therapies have expanded in recent years, leaning on advances in immunology and cancer biology. Therapies, such as phototherapy (PT), immunotherapy, and nanomedicine, have generated great interest due to their unique strengths. For example, nanomedicines are designed to enhance cancer imaging or improve drug targeting,^6^ while immunotherapies are designed to enrich the immune system, increase the specificity and persistence of the therapy, and combat cancer-induced immunosuppression.^7^ Both therapies offer advantages over conventional therapies like radiation and surgery, but they also have disadvantages. Nanomedicine has limited effectiveness in clinical practice,^6^ due to factors such as low delivery efficiency and a lack of tumor models that successfully mimic the human immune response. Similarly, immunotherapy struggles to produce results in clinical trials, an effect credited to factors, such as the immunosuppressive nature of many cancers.^8^

PT is another novel therapeutic option that has increased in utilization for cancer therapy in recent decades. It has its roots in heliotherapy using sunlight or moonlight,^9^ and it began to be used in cancer therapy as early as the turn of the twentieth century.^10,11^ Since then, numerous studies have been conducted to investigate how PT can be used in the treatment of cancer, leading to the emergence of several types of PTs, such as photodynamic therapy (PDT), photothermal therapy (PTT), and photochemical internalization (PCI). Due to the scope of this paper, we focus on these three modalities. Generally, PT possesses high specificity and minimal invasiveness, making it desirable for treating cancer.^12–14^ However, PT does suffer from unique weaknesses, such as shallow penetration in many tissues,^15,16^ lack of oxygen (a required ingredient for PDT and PCI) in hypoxic tumors, and an inability to sufficiently heat large tumors (as designed for PTT).

Due to the weaknesses in different types of monotherapies, some researchers have begun investigating combination therapies to enhance efficiency against cancer.^17,18^ PT is a suitable candidate since it is not cross-resistant with other treatments, meaning it can be used together with other cancer therapies without diminishing their effectiveness.^19–21^ Treatments like nanomedicine and immunotherapy can benefit PT, such as through efficient deliveries of oxygen using nanoparticles to augment the effects of PDT,^22^ combining PTT with stimulants/adjuvants to bolster the effectiveness of the overall immune responses^23^ or utilizing PCI to deliver checkpoint-targeting drugs directly to the cytosol of tumors expressing the checkpoint.^24^ As clinical trials and pre-clinical studies show, there is great potential in combining PT with other types of therapy to produce a more robust and vigorous treatment regimen for cancers.

PT is also an attractive candidate for cancer theranostics, a combination of therapeutics and diagnostics. Many photonics concepts, such as refractive indices, fluorescence, and photon scattering, can be implemented with PT devices or used alongside these therapies. This allows researchers to simultaneously monitor and treat a variety of cancers and more effectively tailor treatments to patients in real-time. In this review, we will briefly discuss some modern therapies, such as nanomedicine, immunotherapies, and PT, as well as diagnostic techniques like surface plasmon resonance (SPR) and fluorescence imaging. We will pay specific attention to photonics-based PTs, particularly their development, applications, advantages, and limitations. We will then examine how the combination of PT with other modern cancer therapies enhances their effectiveness and addresses their shortcomings and how diagnostic technologies are being combined with these therapies to achieve robust theranostics results. Finally, we will discuss future directions and identify areas of interest that could greatly benefit the field of cancer therapy.

## II. MODERN CANCER THERAPIES

### A. Introduction to cancer therapies

While the causes of cancer are multifactorial, it is known that a variety of factors, such as viruses, lifestyle choices, genetics, and environmental factors, can contribute to cancer development.^25,26^ Many cancers are notorious for evading the immune system,^27–30^ leading to a greater need for innovative and effective treatments. Initially, surgery and radiotherapy led the way for cancer therapeutics, but plateauing cure rates due to metastatic disease encouraged the development of chemotherapy.^31^ However, the narrow therapeutic window and the palliative nature of chemotherapy drove research into new options for patients. Herein, we will examine certain modern options for cancer therapy: nanomedicine and immunotherapy, particularly immune checkpoint inhibitors (ICIs).

### B. Nanomedicine

One major drawback of cancer therapies, like chemotherapy, is their inability to discriminate between cancerous and healthy cells, often leading to undesired side effects.^32^ This concern has driven much research into modern cancer therapies, and nanomedicine is no exception.^33^ Specifically, nanomedicine research aims to achieve the following goals: targeted delivery and sustained release of drugs in tissue- and cell-specific manners, enhancement of stability, pharmacokinetics, and bioavailability of therapeutic molecules, and improved cancer imaging for diagnosis.^6^ Due to an explosion of interest in nanomedicine, many therapeutics have been developed and tested for use in humans. One such therapeutic, nanoliposomal irinotecan, completed phase III clinical trials in 2015, successfully extending survival in patients with metastatic pancreatic ductal adenocarcinoma.^34^

Despite the promising results from trials like the one described above, nanomedicine does suffer from some notable deficiencies, which hamper its use as a monotherapy. For example, nanomedicines tend to do well in preclinical settings but lose effectiveness when they are put into clinical practice.^6^ This is due to several factors, especially the lack of efficient tumor delivery, deep intratumoral penetration, effective *in vivo* and *in vitro* models, and scalable production for complex nanomaterials. Additionally, tumors are not simple clumps of cells within our bodies; they are highly complex systems with a variety of mechanisms to defend against our natural immune system. As described by Valerio *et al.*, “The TME (tumor microenvironment) is a corrupted ecosystem independent of the biology of the body. Composition and architecture of the TME are unique and different from any tissue.”^35^ A one-size-fits-all solution cannot exist due to the unique natures of both tumors and immune systems in the patients. Nanomedicine also suffers from safety concerns regarding toxicity and stability issues related to the storage of nanomedicine products.^33^ Because of these issues, van der Meel *et al.* suggests a four-pronged approach to improving nanomedicine translation, which includes proper immunomodulation and combination therapies involving nanomedicine.^36^

### C. Immunotherapy

Immunotherapy is an umbrella term that encapsulates several different types of therapy, all of which aim to guide the immune system to better fighting cancer and tumors.^7^ Immunotherapies include cancer vaccines,^37,38^ adoptive cell transfer,^39,40^ cytokine therapy,^41,42^ adjuvants,^43,44^ ICIs,^45^ and indirect activation of the immune system via oncolytic viruses.^46,47^ Immunotherapies possess several advantages over other types of cancer therapy, such as higher specificity, accuracy, and persistence than traditional therapies^7^ and increased safety profiles due to the ability to customize treatments.^48^ These advantages have driven research and development of immunotherapies, leading to countless discoveries about how our natural immune system works to fight cancer. Higano *et al.* showed that an active cellular immunotherapy known as sipuleucel-T was able to provide survival benefits for patients with advanced prostate cancer.^49^ This treatment involves the use of antigen-presenting cells, which would be activated *in vitro* before being reintroduced to the patient, demonstrating the benefits of adoptive cell therapy. Many of the other treatments above also have great preclinical results, but they are not without their weaknesses and limitations.

While immunotherapies have shown great promise in preclinical settings, many fall short once they enter clinical trials. One major boundary to the success of many immunotherapies is the ability of cancer to suppress the host immune system.^8^ This immunosuppression allows the cancer to evade treatment, ultimately leading to poor response rates to treatment. Additionally, the unique structure of the TME provides challenges for certain types of immunotherapies. For example, chimeric antigen receptor (CAR)-T cell therapy, a promising option for the treatment of B and plasma cell cancers, struggles to treat solid tumors. Glover *et al.* published a review highlighting many of the potential reasons for this challenge, including the physical barrier constructed by the TME preventing CAR-T cells from properly infiltrating the tumor, the loss of the target antigen on tumor cells to prevent targeting by CAR-T cells, and the expression of immune checkpoints by tumor cells to inhibit CAR-T cell function.^50^ These limitations have inspired many researchers to suggest that using immunotherapies in combination with each other or with other therapies may be the key to beating cancer in a wide variety of patients.

### D. Immune checkpoint inhibitors (ICIs)

As mentioned in Sec. II C, immune checkpoints are a prime target for cancer immunotherapies, with ICI providing effective treatment. One strategy which cancer cells employ to avoid the immune system is the expression of certain cellular markers normally used to prevent autoimmunity. These molecules serve to temper the immune system and carefully control the level of activation during an immune response. Seeking to evade the immune system, tumor cells can express large amounts of these checkpoints to avoid detection and elimination by the immune system, leading to an immunosuppressive effect.^51^ Among the checkpoints, programmed cell death protein 1 (PD-1), programmed death-ligand 1 (PD-L1), and cytotoxic T lymphocyte-associated antigen 4 (CTLA-4) are targets initially identified for ICI due to their involvement in immune responses to cancer. PD-1 is expressed on T cells and B cells, so tumor cells express the associated ligand, PD-L1, to inhibit a T cell response. Similarly, CTLA-4 is expressed by T cells naturally to aid in inhibitory activities, so tumors will express the associated ligand, B7, to decrease the effectiveness of T cells against the tumor. Therefore, immune checkpoint inhibitors (ICIs) targeting these three (PD-1, PD-L1, or CTLA-4) have successfully translated to clinical use to induce a robust T cell response to the tumor.

The first FDA-approved ICI was ipilimumab, targeting CTLA-4, in 2011.^52^ Since then, multiple ICIs against PD-1, such as pembrolizumab^53,54^ and nivolumab,^55,56^ have been approved by the FDA for use in the treatment of certain cancers, with the list of targeted cancers growing over time.^52^ Hodi *et al.* studied the effects of ipilimumab in combination with a glycoprotein 100 against advanced metastatic melanoma.^57^ In this study, the investigators found that ipilimumab led to significantly improved survival against the control group of glycoprotein 100 only. Additionally, they found that the inclusion of glycoprotein 100 did not significantly alter the effects of ipilimumab, suggesting that the main benefit of the treatment comes from the ICI itself. In 2015, Robert *et al.* published a study on the use of nivolumab against advanced metastatic melanoma.^58^ Compared to dacarbazine, a chemotherapeutic agent used in treating melanoma,^59^ Robert *et al.* found that nivolumab provided significant improvements to overall patient survival and progression-free survival.

Despite these promising results, however, ICI faces challenges in anti-tumor efficacy as a monotherapy in some tumors. For example, certain solid tumors are resistant to ICI, a facet attributed to the heterogeneity of the tumors and their associated microenvironments.^51^ Even in cases where ICI proves to be effective initially, patients may develop a resistance to the therapy,^60–63^ leading to further decreased overall efficiency in clinical applications. Furthermore, immune-related adverse events, while not necessary to produce satisfactory results from ICI, can affect certain patients without a clear predictor to their presence before treatment.^64,65^ While these events are generally reversible, some are not, limiting the clinical applications of ICI until the mechanism of these immune-related adverse events is more clearly understood. Finally, many patients see no benefit from ICI, developing resistance to the treatment due to numerous potential factors.^63^ Thus, single-agent ICI has limitations, resulting in many researchers investigating alternatives to treating cancer.

## III. PHOTOTHERAPY

### A. An introduction to phototherapy

PT initially began to attract attention as a cancer therapy following its approval in Canada as a clinical cancer therapy in the early 1990s.^66^ In the following decades, PT has become an option for a variety of cancers due to several key features of PT. Several different forms of PT for cancer exist, primarily PDT, PTT, and PCI. Despite their differences, PDT, PTT, and PCI all benefit from high specificity and minimal invasiveness.^12–14^ Additionally, PDT, PTT, and PCI possess little cross-resistance with other treatments, meaning they can be used in conjunction with other forms of cancer therapy to enhance these treatments without diminishing their effectiveness.^19–21^ These combination modalities will be explored in further detail later in this review. In addition to therapy, numerous imaging techniques and devices have been developed, leading to the emergence of cancer theranostics, or the combination of cancer therapeutics and cancer diagnostics. These evolving technologies will be discussed further later in this review.

### B. Photodynamic therapy (PDT)

**FIG. 1.**
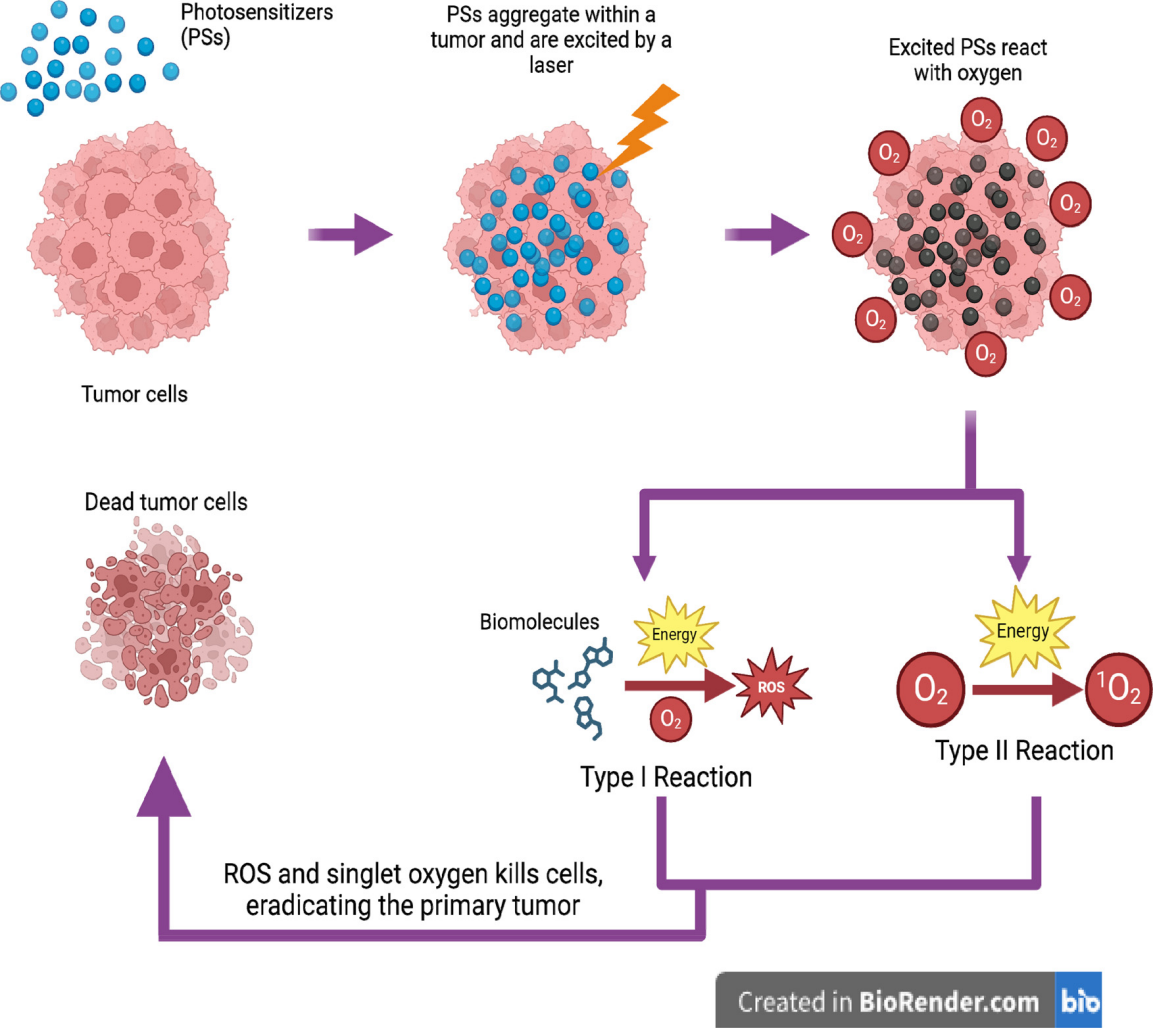
Functions and principles of photodynamic therapy. Photodynamic therapy is a technique used to eliminate primary tumors using light and a special class of molecule known as a photosensitizer (PS). These PSs use light energy to react with oxygen to form radical oxygen species (ROS) from biomolecules or oxygen itself through type I or type II photochemical reactions, respectively. These ROSs, like singlet oxygen and free radicals, mediate cellular toxicity (figure created with BioRender).

PDT is a minimally invasive and clinically approved procedure that has shown promise in treating a variety of cancers, such as gastrointestinal cancers,^67^ bile duct cancer,^68^ and bladder cancer.^69^ PDT utilizes a photochemical reaction that generates reactive oxygen species (ROSs) to kill tumor cells. In addition, these ROSs damage the tumor's vascular structure and initiate an immune response against the tumor cells.^70^ To accomplish this reaction, PDT requires three key elements: oxygen, light, and a photosensitizer (PS).^71^ As shown in Fig. 1, PSs are activated by light to react with oxygen, generating ROSs.^72^ PSs absorb energy from the light source, transitioning from its ground state to an excited state. While in this excited state, the PS reacts with biomolecules, such as proteins or oxygen, in a type I or type II photochemical reaction, respectively. Both reactions produce ROSs, which then damage the tissues around them.

Regardless of which type of reaction generates the ROSs, physicians and researchers seek to optimize the amount of ROSs produced relative to the amount of light and PSs used. One way they can do this is by identifying which factors influence the production of ROSs within a patient. Effective penetration depth is the depth at which the intensity of light has decreased to 1/e (approximately 37%) of the intensity at the surface of the media.^73^ This is a crucial factor in determining how efficiently ROSs will be produced. This variable is especially important in tumors that are large, such as later stage primary tumors, or tumors that are deep within a patient, as tumors that fit one of these profiles may not be completely treated if the effective penetration depth is less than the depth at which the tumors are located or to which the tumors extend. This depth can be determined using an equation from Plaetzer *et al.*, shown below. Their original equation was generated using the Beer–Lambert Law and correlates intensity at a specific penetration depth ( $I_{x}$) with the intensity at the media interface ( $I_{0}$), the penetration depth ( x), the absorption coefficient ( $\alpha_{\text{abs}}$), and the scattering coefficient ( $\alpha_{\text{sca}}$) as follows:^74^

$$I_{x}=I_{0}e^{-\left(\alpha_{\text{abs}}+\alpha_{\text{sca}}\right)x}.$$ (1)

We can solve their equation for x [Eq. (2): Modification of Beer–Lambert law]

$$x=-\frac{\text{ln}\left(\frac{I_{x}}{I_{0}}\right)}{\left(\alpha_{\text{abs}}+\alpha_{\text{sca}}\right)}.$$ (2)

We can further simplify this equation because, for the effective penetration depth, the ratio of $I_{x}$ to $I_{0}$ is 1/e. Therefore, to find the effective penetration depth, we substitute in the absorption and scattering coefficients of the media being investigated to determine the final value of x [Eq. (3): Simplified modification of Beer–Lambert law)

$$x=\frac{1}{\left(\alpha_{\text{abs}}+\alpha_{\text{sca}}\right)}.$$ (3)

Using the above-mentioned equation and the known coefficients for absorption and scattering for the material, we can estimate the effective penetration depth. However, factors, such as the wavelength used, can also affect the effective penetration depth, as wavelength, penetration, and energy share a relationship. In general, longer wavelengths can more easily penetrate tissue but carry less energy. Therefore, higher wavelengths that can penetrate deep into tissue may not have the energy to excite oxygen molecules and begin the ROS generation process. Meanwhile, shorter wavelengths with sufficient energy may not penetrate deep enough into the tissue. As a result, many researchers have focused on creating more ideal PSs to increase the efficiency of PDT, balancing penetration with energy carried. For example, second-generation PSs were developed to increase the formation of singlet oxygen ROSs, while third-generation PSs should aim to possess higher tumor tissue selectivity and longer wavelength activation.^75^

Noh *et al.* developed a PS that targets mitochondria. Typically, mitochondria are receptive to cationic agents due to its negative transmembrane potential; this effect is further enhanced by cancerous mitochondria, whose transmembrane potential's magnitude is further increased.^76^ To take advantage of this, Noh *et al.* developed a unique PS from heptamethine cyanine dyes. This PS was conjugated to a triphenylphosphonium derivative for mitochondrial targeting and brominated to enhance ROS generation. They showed that the PS had increased mitochondrial targeting and ROS generation.^76^

Another approach to improving PDT is altering how PSs absorb and emit energy. Aggregation-induced emission is a term used to describe the phenomenon of certain substances exhibiting greater emission when molecules aggregate compared to individual molecules of that substance.^77^ When applied to PDT, aggregation-induced emission PSs exhibit favorable properties and can be used in a variety of functions, such as disease diagnosis, cancer cell ablation, bacterial infection treatment, image-guided surgery, and image-guided therapy.^78^ These and other modifications to PSs allow for more robust and targeted therapies that enhance PDT in numerous ways.

### C. Photothermal therapy (PTT)

**FIG. 2.**
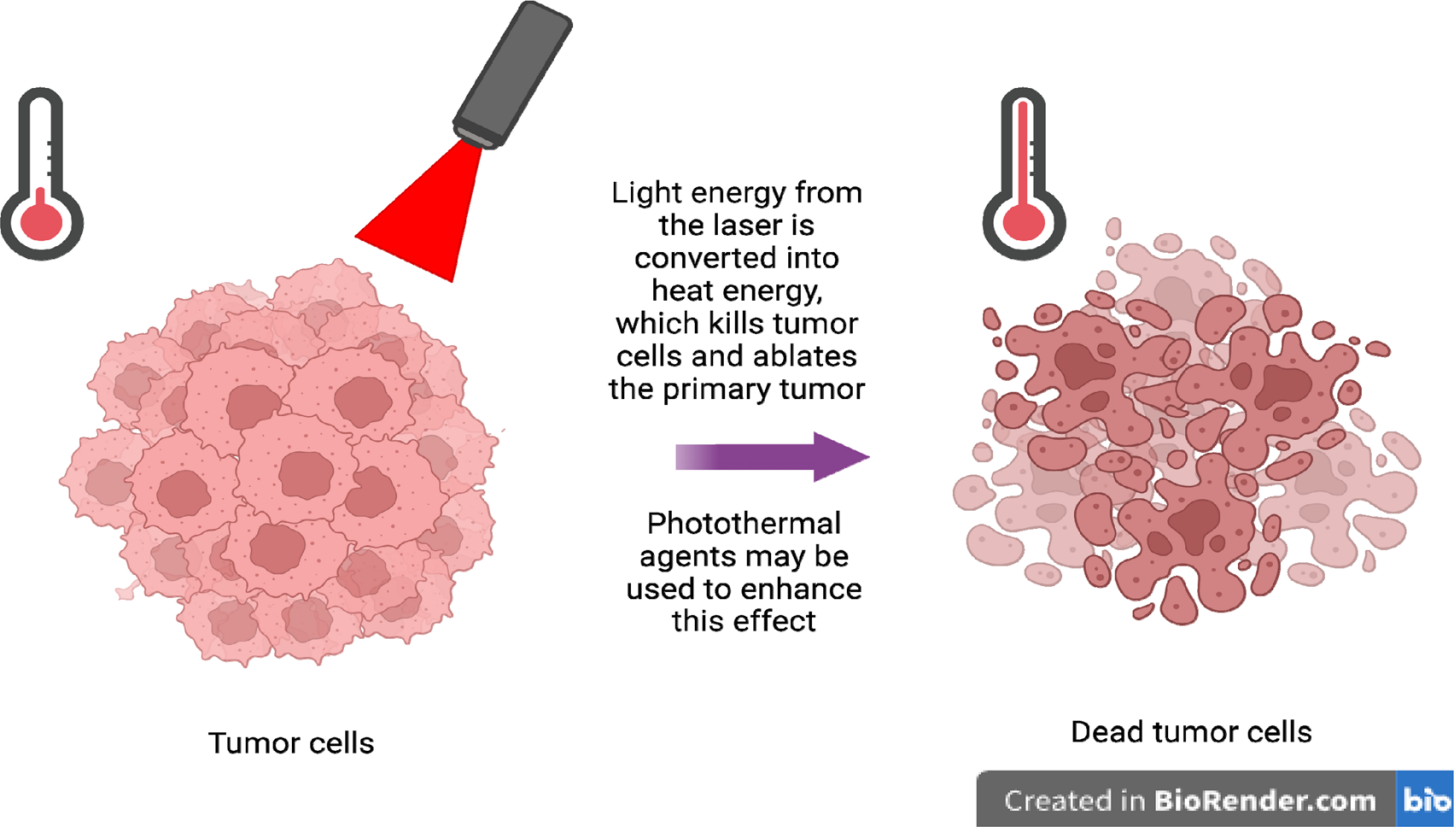
Principles of photothermal therapy. Photothermal therapy is a technique used to ablate primary tumors using light. Light energy are converted into heat to which tumor cells are sensitive. This increase in heat kills tumor cells, leading to the ablation of the tumor. Compounds known as photothermal agents can be used to enhance this effect (figure created with BioRender).

PTT's minimal invasiveness and high selectivity have made it an appealing therapeutic option for a variety of cancers.^13,79,80^ PTT targets tumors with a laser, converting the light energy into heat. As shown in Fig. 2, PTT causes the targeted tissues to increase in temperature, eventually leading to cell death. Cancer cells are especially susceptible to heat, making PTT an effective means of ablating tumors.^79,80^ PTT is often used with near-infrared (NIR) light, as it can penetrate deep tissues without causing major damage to healthy tissues.^81,82^ Additionally, PTT can utilize photothermal agents (PTAs), such as indocyanine green dye (ICG) and porphyrins, to more efficiently convert light to heat. Physicians can combine PTAs with NIR to create therapies that effectively eliminate tumors with minimal damage to healthy tissue. To best accomplish this, an ideal PTA will efficiently convert light energy to heat, allowing for the longer wavelengths of NIR light to be utilized and, thus, penetrate deeper into tumors. Therefore, when designing new PTAs, researchers will consider their conversion rates of light energy to heat. By maximizing this conversion rate, researchers can create highly efficient PTAs, allowing physicians to administer them in smaller doses, thereby reducing side effects. To determine the conversion rate for a PTA, we can use the following equation, where η is the photothermal conversion efficiency, Q is the generated thermal energy, and E represents the total energy of the incoming light [Eq. (4): Conversion rate of photothermal agents]

$$\eta =\frac{Q}{E}=\frac{\text{cm}\Delta \text{T}}{\text{pst}}.$$ (4)

Above, we converted Q and E using their respective equations, where c is the specific heat of the PTA, m is its mass, ΔT is the temperature increase in that PTA, p is the power density of the light source, and s and t represent the irradiated surface area and time for the light source focused on the PTA.^83^ Factors, such as heat loss and heat transfer through radiation, conduction, and convection, will play a role in determining or designing the optimal PTA. Some of these factors, such as specific heat, are influenced by the material the PTA is made from, while others, like heat transfer, are influenced more by the geometry of the PTA. Therefore, it can be helpful for researchers to use this equation to determine how efficient a PTA is. They can then design PTAs with high efficiency by maximizing the heat generated or minimizing the light energy consumed.

Zhou *et al.* studied the effects of PTT on a murine mammary tumor model, EMT6.^84^ While the team was primarily looking at the effects of a nanomaterial in combination with PTT, they did find that PTT alone increased survival and decreased tumor burden in mice compared to the control groups.^84^ Chen *et al.* studied the effects of PTT in combination with an immunostimulant for cancer treatment^85^ and reported their findings on how ICG (a PTA for enhancing light around 800 nm), glycated chitosan (GC) (an immunostimulant), and laser irradiation (PTT), affect a metastatic breast cancer model in rats. They found that PTT led to increased survival compared to the control group and the ICG alone group.^85^ Since then, numerous papers on PTT in combination with other treatments have been published.^86–91^

### D. Photochemical internalization (PCI)

**FIG. 3.**
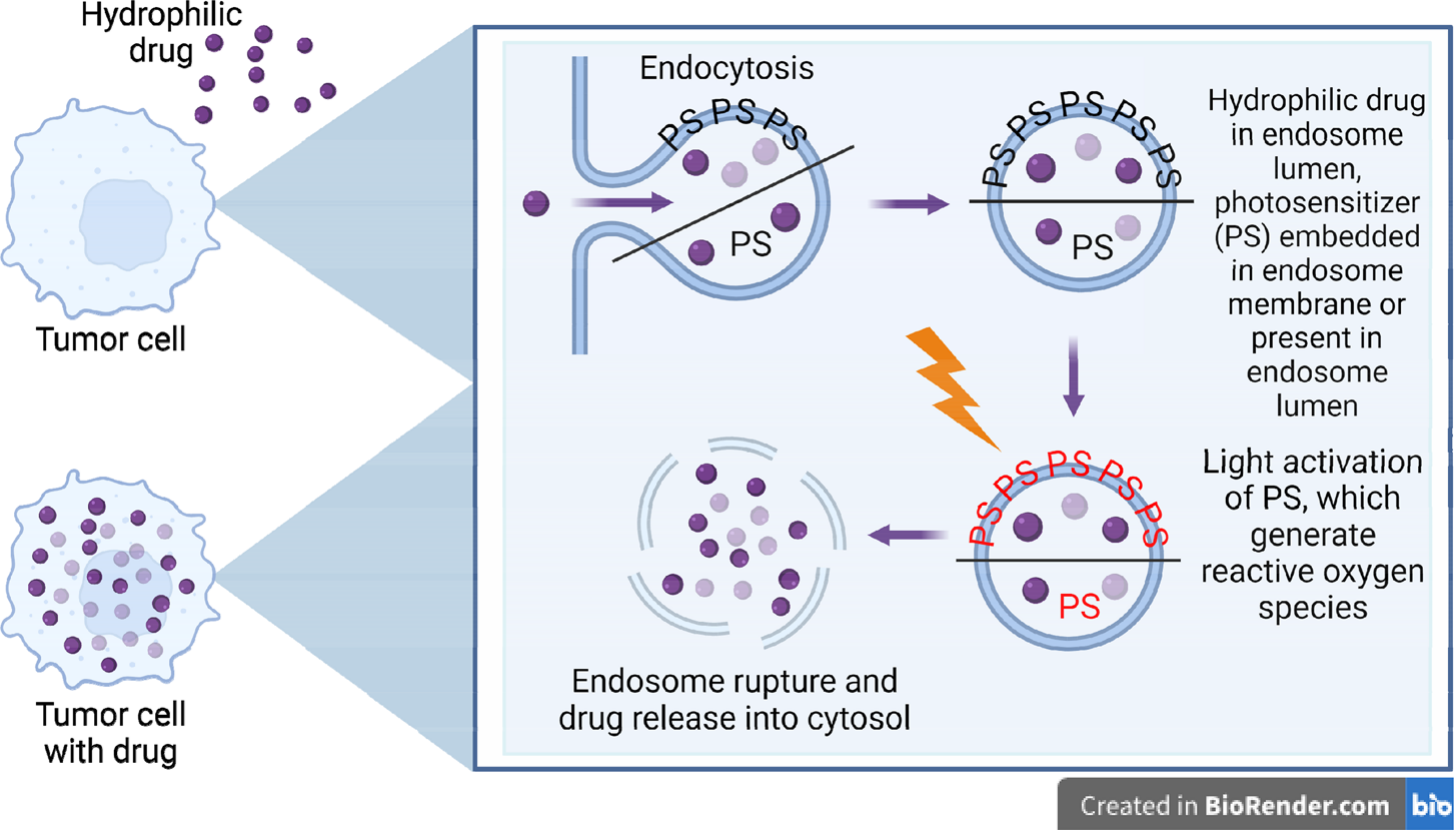
Principles of photochemical internalization. Photochemical internalization is a technique used to facilitate the transport of molecules, particularly hydrophilic ones, into specific areas of the body like tumors. The molecules in question are taken up by cells via endocytosis, along with amphiphilic photosensitizer which embeds itself into the endosomal membrane. Once the molecules have been internalized, light is used to disrupt the endosomal membrane, leading to the release of the macromolecule throughout the cell (figure created with BioRender).

PCI is a novel PT, originally developed by Berg *et al.* in 1999 when the team was attempting to address the issue of inefficient macromolecule transfer to the cytosol.^92^ In its first publication, PCI was described as a “novel technology… found to efficiently deliver type I ribosome-inactivating proteins, horseradish peroxidase, a p21^ras^-derived peptide, and a plasmid encoding green fluorescent protein into cytosol in a light-dependent manner.”^92^ The principle behind PCI is demonstrated in Fig. 3, where the use of certain PSs localized to endosomes and lysosomes of cells. The most efficient PSs for PCI tend to be those with an amphiphilic structure possessing a hydrophilic structure that inhibits cell membrane penetration.^14^ Once these PSs are taken up by cells, they are exposed to light, which generates ROSs that destroy the membranes of these vesicles and deposit their contents into the cytosol. Combined with specific macromolecules, this ingenious technique allows for the efficient transport of these molecules into specific cells. PCI is the ideal method for delivering therapeutics incapable of breaching cellular membranes, as its unique action maximizes the intracellular delivery of these therapeutics.^93^

Because PCI uses the same basic principle as PDT, it also possesses the same effects that make PDT desirable for fighting cancer: direct killing of cancer cells, damage to the tumor vasculature, and potential activation of the immune system. Additionally, PCI possesses a fourth effect, allowing it to carve a niche for itself amongst other PTs: the ability to control the release of the contents of endocytic vesicles directly into the cytosol through photochemical mechanisms.^94^ It is this key feature that has driven research into PCI, such as Jørgensen *et al.*'s work with biodegradable peptide carriers^95^ and Berg *et al.*'s further work into the development of new and more efficient PSs for PCI.^96^

While PCI is commonly used with drug delivery, it has also deployed in the delivery of genes for cancer therapy, especially with nonviral genes, even when aided by cationic polymers, due to biological membranes.^97^ PCI offers a method of crossing these membranes through endocytosis. These genes can then be delivered directly into the cytosol via irradiation. To demonstrate this idea, Xu *et al.* developed a star-shaped polypeptide, embedding a PS for later PCI implementation.^97^ This polypeptide showed significantly higher transfection rates compared to linear analogues, and the PCI irradiation induced an almost complete endosomal release of the DNA cargo, leading to further increases in transfection efficiency.^97^ Moreover, a 2014 paper by Wang *et al.* analyzed PCI's potential in glioblastoma as a delivery mechanism for genes.^98^ In this study, the team attempted to use PCI as part of a gene delivery system to transfect glioma cells with a “suicide gene” that would convert a nontoxic antifungal agent into a chemotherapeutic drug. The transfection of this gene was enhanced by PCI and resulted in antitumor effects in the presence of the original nontoxic agent, demonstrating the glioma cells' new ability to convert the antifungal to the chemotherapeutic and inhibiting glioblastoma spheroid growth.^98^ These results were verified by compared to PDT therapy without the antifungal agent. Therefore, PCI represents a burgeoning field of cancer therapeutics, serving as a promising new avenue for cancer therapy.

## IV. COMBINATION THERAPIES

### A. Shortcomings of PDT, PTT, and PCI

In general, PT has great promise as a therapy for various types of cancer. Its minimal invasiveness and high selectivity give it the potential to be highly effective against tumors, though it is still limited by certain weaknesses. For example, one of the greatest benefits to PT is its selectivity due to the need for light to activate its therapeutic effects. However, light cannot penetrate further than a few millimeters into tissues.^15^ This is because different components of tissue, such as water, melanin, and hemoglobin, affect the overall penetration of light by absorbing longer and shorter wavelengths of light.^16^ Additionally, while longer wavelengths can penetrate further into a tissue, they carry less energy and may be unable to initiate reactions necessary for ROS generation. Therefore, there is a window of opportunity for therapeutic effects, between 600 and 800 nm for PDT.^99^ This range still delivers sufficient energy to activate PT while successfully penetrating deep enough to reach the tumor. However, tumors exist which fall outside of the effective depth of this range, leading to a need for advancements to address this concern. Finally, PT often struggles to target metastases due to the limited depth and localized nature of PT. Given metastasis is responsible for the high mortality of many cancers,^100^ this downside of PT has the interest of many researchers who are looking to address it using combination therapies.

Beyond the general limitations associated with PT, each type of PT has specific treatment-related weaknesses. For example, PDT requires oxygen in most of its applications to work, utilizing the oxygen-dependent type II reaction to generate ROSs.^101^ While this gives PDT the ability to produce potent antitumor responses, it leads to a glaring weakness: hypoxia. If a certain PDT treatment utilizes these type II reactions and is introduced to or produces a hypoxic environment, such as those in many solid tumor TMEs, the efficacy of this treatment is greatly reduced. Since PCI operates on similar processes to PDT, it suffers from lower efficacy in hypoxic environments.

PTT, while free of the oxygen dependence of PDT and PCI, carries its own limitations. Since the primary mechanism of tumor cell killing for PTT comes from thermal ablation, risks involving the high temperatures must be considered when weighing the risks and benefits of PTT for a patient. For example, surrounding tissues may be damaged while heating a tumor, leading to an unintended immune response from the patient. Additionally, PTT has limitations in providing deep heating for larger tumors. If tumors survive the initial bout of heating, they can develop a resistance to thermal ablation, limiting future uses of this therapy in that patient.^102^

**FIG. 4.**
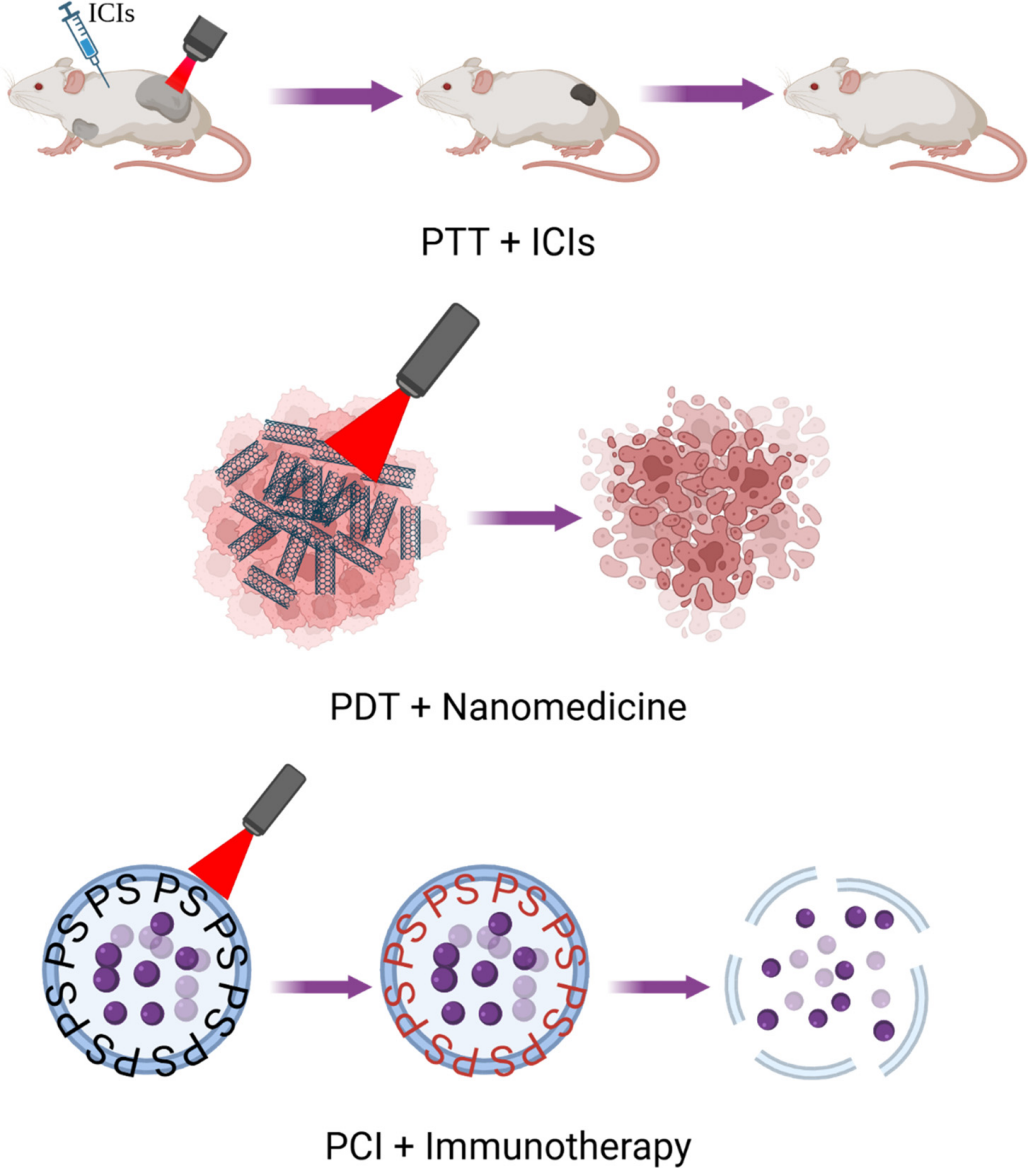
Combination therapies overview. Phototherapy serves as an excellent combination partner for a variety of other therapies, such as nanomedicine and immunotherapy. Examples include photothermal therapy (PTT) combined with immune checkpoint inhibitors (ICIs), photodynamic therapy (PDT) combined with nanomedicine, and photochemical internalization (PCI) combined with immunotherapy (figure created with BioRender).

These limitations, summarized in Table I, drive research into the combination of PT and other therapies. This research approach involving combination therapy will likely be more successful long term. We have briefly summarized combination therapies in Fig. 4.

**TABLE I.** Summary of limitations of monotherapeutic PT. PT—phototherapy, PDT—photodynamic therapy, PTT—photothermal therapy, and PCI—photochemical internalization.

Type of PT	Limitations
PDT, PTT, PCI	Components of tissue absorb certain wavelengths of light, affecting its penetration depth
PDT, PTT, PCI	Deep tumors cannot receive sufficient energy due to the relationship between wavelength, energy, and penetration depth
PDT, PTT, PCI	PT does not effectively target metastasis, a major source of mortality in many types of cancer
PDT, PTT, PCI	PT can fail to completely eradicate a tumor due to factors like heterogeneous light distribution and uneven drug uptake, leaving behind viable cells, leading to recurrence
PDT, PTT, PCI	PT has limited systemic effects, often failing to induce strong immune memory
PDT, PCI	PDT and PCI require oxygen to work, so hypoxic tumor environments lower their efficacy
PDT, PCI	Photosensitizers often have poor tumor selectivity, slow clearance, or suboptimal pharmacokinetics
PTT	Surrounding tissues can be damaged by high temperatures induced by PTT
PTT	Deep heating of tumors is difficult to achieve
PTT	Residual tumor cells after initial heating can develop resistance to thermal ablation
PCI	PCI possesses limitations on the type of cargo it can utilize, restricting its usage in cases where the target drug or molecule is incapable of being transported via PCI

### B. PT + nanomedicine

**FIG. 5.**
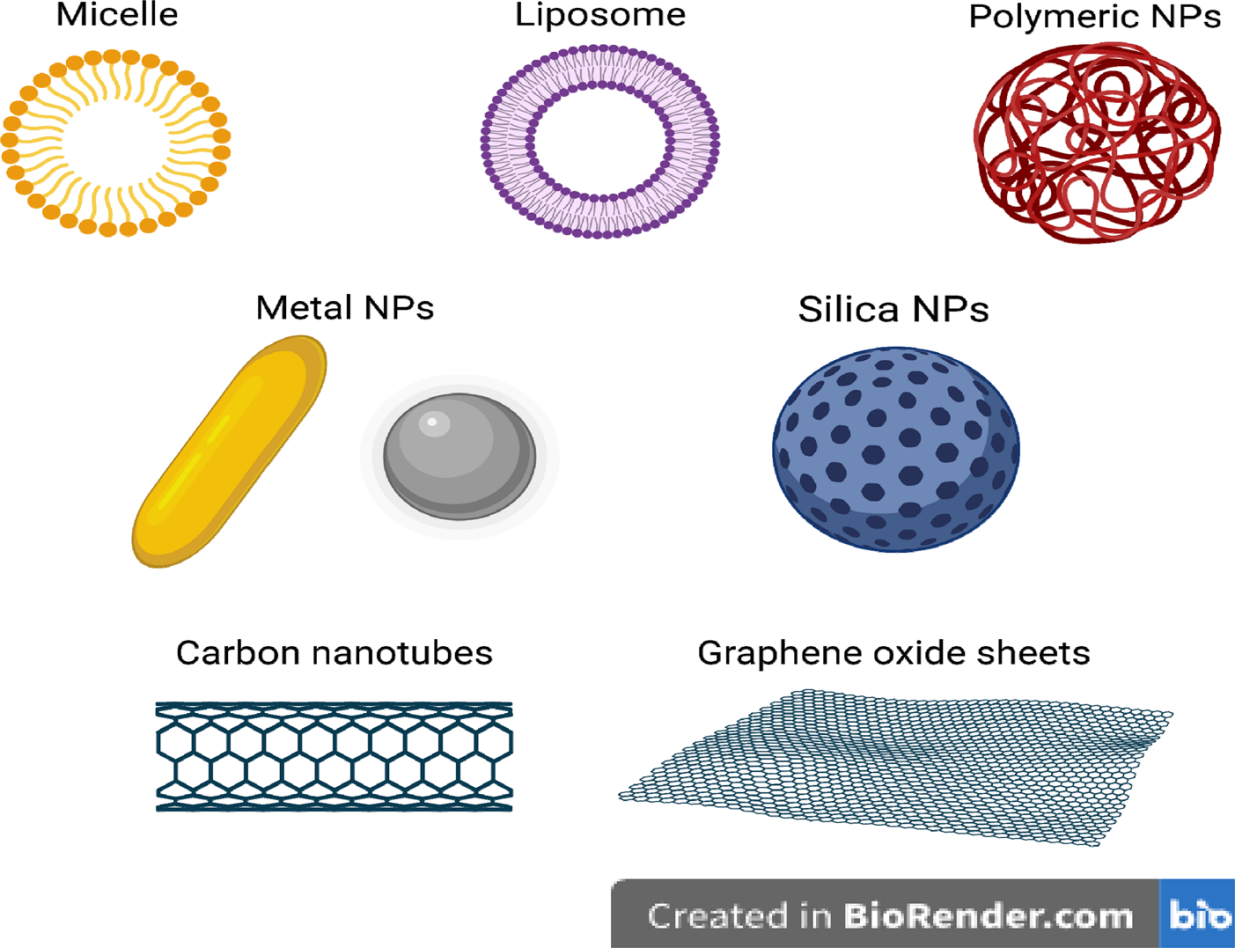
Common examples of nanomaterials for phototherapy. Examples include micelles, liposomes, polymeric NPs, metal NPs, silica NPs, carbon nanotubes, and graphene oxide sheets (figure created with BioRender).

Nanomaterials and PT synergize well due to nanomaterials' ability to more efficiently deliver necessary adjuncts for PT. For example, a nanomaterial may be designed to more efficiently convert light to heat when loaded with tumor-targeting agents, increasing the efficiency of PTT. Alternatively, PDT and PCI, needing both a photosensitizer and oxygen to efficiently kill tumors, can make use of nanomaterials to ferry these necessary compounds directly to a tumor. Furthermore, photoreactive nanomaterials are a key focus for photonics related cancer research, serving a theranostic role. The form of nanomaterials determines how effective they are in these functions. For example, the nanomaterial's surface chemistry and functionalization through processes like PEGylation and coating with targeting ligands influence its biodistribution and tumor selectivity. Additionally, core nanomaterial properties, such as the size, shape, and composition, dictate its phototherapeutic performance, and overall biocompatibility and clearance profiles must be considered for clinical translation. Figure 5 illustrates common nanomaterials, while Table II presents the results of the combination of nanomedicine and PT.

**TABLE II.** Results of the combination of nanomaterials and PT. CaCO_3_-MnO_2_—calcium carbonate manganese, NP—nanoparticles, PDT—photodynamic therapy, TiO-ZSN—titanium oxide coated zinc-based porphyrin self-assembled nanocrystals, TP-PDT—two-photon PDT, GO—graphene oxide, NS—nanosheets, NIR-PTT—near-infrared photothermal therapy, GQD—graphene quantum dots, SWNT—single-walled carbon nanotubes, GNSt—gold nanostars, MP-NbC—mesoporous niobium carbide, and PCI—photochemical internalization.

Tumor model species	Treatment	Major results	Reference
Lewis lung tumors in mice	CaCO_3_-MnO_2_ NP + PDT	Combination improves tumor hypoxia and shows enhanced PDT effect	105
4T1 tumors in mice	NP + PDT	Combination shows improved PDT efficacy, enhanced immune response, destruction of primary tumors, and suppression of distant tumors	22
HeLa cells *in vitro*	TiO-ZSN + TP-PDT	Combination induces cell death and has applications in NIR fluorescent imaging	106
HeLa cells *in vitro*	GO-NS + NIR-PTT	Combination reduces viability of cells	107
4T1 cancer cells *in vitro*	GQD + NIR-PTT	Combination demonstrates higher cancer killing efficacy	86
MCF7 cancer cells *in vitro*	SWNT + NIR-PTT	Combination demonstrates enhanced PTT effect and leads to a selective cancer therapy	109
EMT6 cancer in mice	SWNT + NIR-PTT	Combination shows greatly enhanced photothermal destruction of tumors	110
4T1 tumors in mice	Reduced-GO + PTT	Combination reduces tumor volume and increases survival	87
Hey tumors in mice	GNSt + NIR-PTT	Combination significantly decreases tumor growth, with complete regression in 75% of treated mice	111
U87 *in vitro* and *in vivo*	MP-NbC NS + NIR-PTT	Combination significantly inhibits tumor growth	112
HeLa cells *in vitro*	Drug-loaded NP + PCI	Combination possesses targeted and enhanced cellular death	113
MCF-7 cells *in vitro*	Drug-loaded NP + PCI	Combination provides potent initial and lasting cellular toxicity	114

#### 1. PDT + nanomedicine

Nanomaterials can be customized to carry a variety of payloads, such as drugs or oxygen. This unique method of enhancing PDT allows for the modulation of the TME during treatment. One way this can be achieved is with hemoglobin, a natural oxygen carrier. Hemoglobin can be encapsulated within a nanoparticle (NP) to deliver oxygen directly to the tumor, as shown by Zhou *et al.*^103^ In addition, some recently developed nanomaterials can reduce the aggregation-caused quenching effect, which is caused by high concentrations of PSs, severely weakening their optical properties.^104^ Liu *et al.* designed a NP platform and tested its effects on enhancing PDT both *in vitro* and *in vivo.*^105^ The study showed that their manganese-calcium carbonate NP system improved the hypoxic TME of a lung cancer model, induced an antitumor immune response, and significantly increased the therapeutic effect in a murine model.^105^

In 2018, Chen *et al.* developed a hybrid protein oxygen NP to attempt to relieve tumor hypoxia and enhance PDT.^22^ Using a 4T1 tumor model, the team found that their NPs increased the oxygenation of tumors on their own and enhanced the efficacy of PDT through the reduction of both primary tumors and distant metastases and the triggering of potent antitumor immune responses.^22^ Liu *et al.* designed a self-assembling zinc-based porphyrin nanocrystal coated with titanium oxide for use in two-photon PDT.^106^ The team reported that their nanocrystals induced a cytotoxic effect in HeLa cells when treated with a laser, effects which were absent in the laser only group, and that their nanocrystals could be used in NIR fluorescent imaging for cancer.^106^

#### 2. PTT + nanomedicine

The use of nanomaterials in PTT is growing every year, as numerous labs are using nanomaterials to enhance the effects of PTT with promising results. Carbon is a commonly used base for many nanomaterials. From graphene oxide nanosheets (NSs)^107^ and graphene quantum dots^86^ to magnetic carbon NPs^108^ and single-walled carbon nanotubes (SWNTs),^109^ many papers have been published on the use of carbon as a material for a variety of nanomedical uses. In 2009, Zhou *et al.* investigated the use of SWNTs conjugated with folate (folate-SWNTs) and combined with PTT against a mammary carcinoma model.^110^ The team found that their folate-SWNTs destroys significantly more tumor cells via PTT while sparing nontargeted cells.^110^ Reduced graphene oxide also has uses in enhancing PTT, activating as an immune adjuvant. Zhou *et al.* analyzed how a nanosystem built from reduced graphene oxide would affect the therapeutic efficacy of PTT against a 4T1 orthotopic tumor model, finding the nanosystem significantly reduced tumor volume and increased survival.^87^

**FIG. 6.**
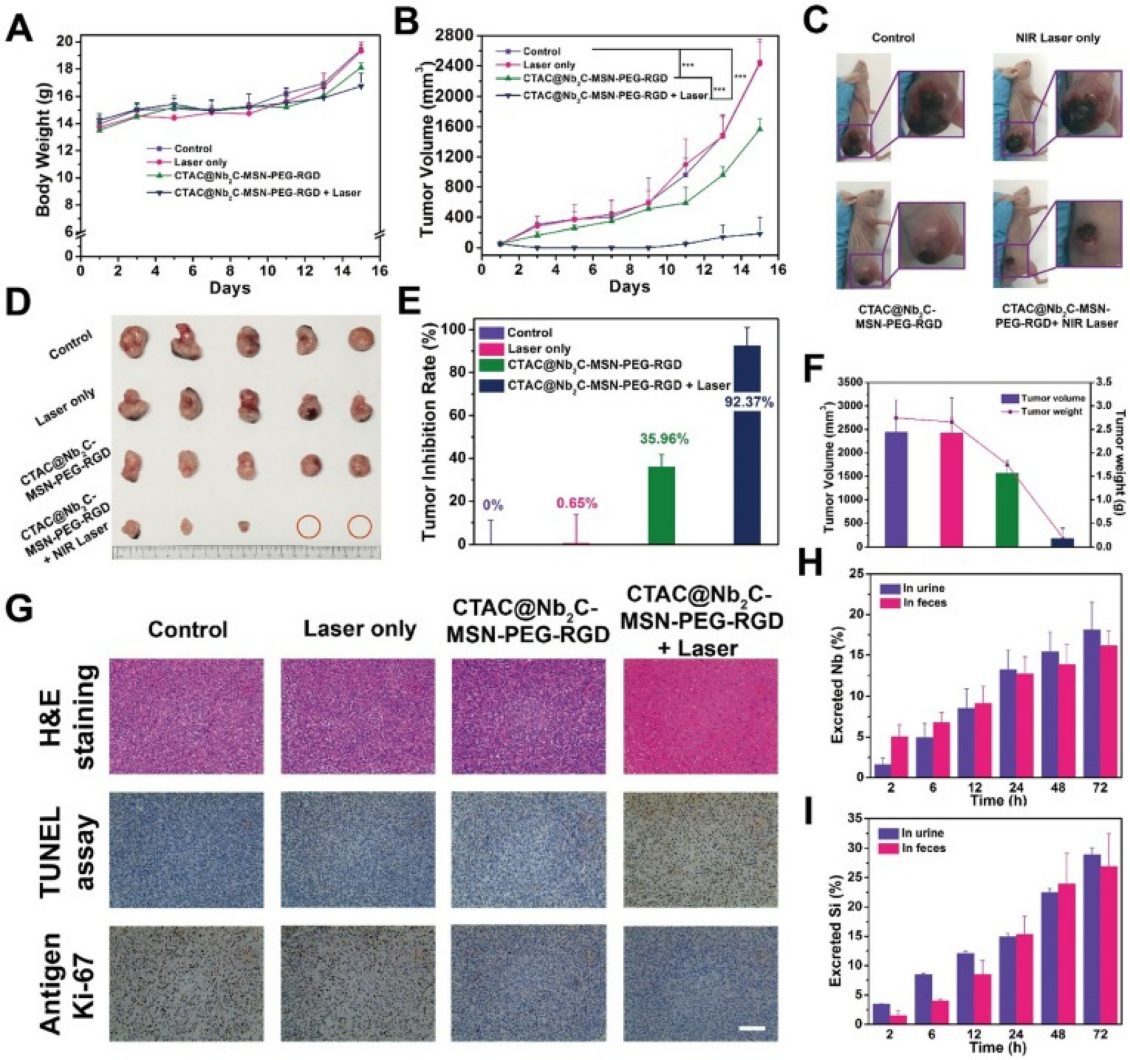
*In vivo* enhanced chemotherapy/PTT. (a) Time-dependent body-weight curves and (b) tumor-volume curves of U87 tumor-bearing mice in different groups (control group, CTAC@Nb_2_C-MSN-PEG-RGD only group, laser only group, CTAC@Nb_2_C-MSN-PEG-RGD combined with laser group). (c) Digital photos of U87 tumor-bearing mice and their tumor regions after varied treatments at day 15 post treatment and (d) isolated tumor tissue after different treatments. (e) Tumor-inhibition rate and (f) the tumor volumes/weights of isolated tumors from each group at day 15 post treatment. (g) H&E staining, TUNEL staining and Antigen Ki-67 immunofluorescence staining in the tumor region of each group at day 15 post treatment. Scale bar: 50 *μ*m. Accumulated (h) Nb excretion and (i) Si excretion after intravenous injection of CTAC@Nb_2_C-MSN-PEG-RGD at different time points (2, 6, 12, 24, 48, and 72 h). Reproduced with permission from Zhou *et al.*, J. Biomed. Opt. **14**(2), 021009 (2009). Copyright 2009 authors, licensed under a Creative Commons Attribution (CC BY) license.

Other materials can be utilized in nanomaterial applications, such as gold and niobium carbide. You *et al.* studied the effects of customized golds NPs on the efficacy of PTT.^111^ These hollow NPs were loaded with a chemotherapy drug and designed to target a known overexpressed receptor, EphB4, on the cell membrane of certain types of tumors. They found that the NPs showed an increased uptake in tumor cells positive for this receptor.^111^ Additionally, the gold NPs significantly enhanced the effect of PTT on tumor ablation and clearance, resulting in six of the eight mice in the laser + NP treatment group clearing their tumors completely.^111^ In a 2018 study, Han *et al.* designed a mesoporous coating for niobium carbide NSs and investigated how this coating enhanced or diminished the NS's therapeutic capabilities.^112^ As shown in Fig. 6, the NS synergized with the laser treatment to more efficiently inhibit tumor growth in a murine neuroglioma model than the laser or NSs alone.^112^ Combined, these results, as well as many others, show the potential of the combination of nanomedicine with PTT.

#### 3. PCI + nanomedicine

Like PDT, PCI heavily relies on oxygen to generate its effect, leading to a reliance on the presence of this vital element, and a weakness to hypoxic regions commonly found in certain cancers. However, just as PCI possesses a unique advantage over PDT, its combination with nanomaterials offers unique perspectives on cancer therapy. To this end, Pasparakis *et al.* constructed a polyacetal and PEG NP capable of degrading upon exposure to certain wavelengths of light.^113^ While only presenting *in vitro* results, the team successfully showed that their system possesses significantly higher cellular uptake of the NPs via an endosomal pathway and significantly higher death rates for groups with NPs irradiated by a laser compared to other control groups.^113^ Panagiotakis *et al.* synthesized several novel conjugates of a cyclic carbohydrate oligomer and a PS, all of which self-assembled into small, stable NPs in water.^114^ They then loaded these NPs with an anticancer drug and tested the efficacy of PCI on their model. The team found that their NPs possessed low toxicity in lightless environments and would cause considerable, albeit transient, toxicity on their own when exposed to light; they also found the resulting PT-induced internalization, when combined with chemotherapeutics, would generate strong initial toxicity as well as a lasting impact on cell proliferation, demonstrating PCI-related uptake of the drug.^114^

#### 4. Insights on PT + nanomedicine

Nanomedicine serves as an excellent combination candidate for PT, enhancing the effects of each other and compensating for the weaknesses of either. For example, NPs can be engineered to convert light energy into heat, allowing both PTT and PDT to be used simultaneously. This allows physicians and researchers to carefully craft treatments and take advantage of these powerful techniques while saving time. Additionally, NPs offer stable and targeted delivery of PSs to tumors, reducing the likelihood of off-target effects and increasing efficacy of PDT in that model. Furthermore, if more PSs can be delivered to the tumor site, the amount needed to be injected is reduced, meaning the chances of local and systemic toxicity from PSs are reduced in patients. Certain nanomaterials allow for deeper tissue penetration of light, either by more efficiently converting light energy or by enabling the use of wavelengths that naturally penetrate deeper, such as near-infrared light. Finally, nanomaterials can more easily overcome biological barriers which may prevent the transport of PSs to tumors, allowing for a broader range of treatment options for those types of cancer. Nanomaterials also can ferry other important molecules, such as oxygen, a vital component of PDT and PCI, which is often lacking in tumors.

When investigating nanomaterials for use in conjunction with PT, the material properties of these nanomedicine agents should be carefully considered. For example, nanomaterials used in combination with PTT should possess a high photothermal conversion rate at longer wavelengths, allowing PT utilizing these longer wavelengths, such as PT for deep tumors, to be more effective. This rate can be determined by factors, such as the NP's size, shape, and composition. Size also plays a role in tumor accumulation and immune clearance, along with surface charge of the nanoparticles.^115^ Furthermore, these properties can affect the biocompatibility of the nanomaterial, a key factor when choosing nanomedicine candidates. These considerations make materials like graphene oxide and gold nanorods preferable due to their high photothermal conversion rates. Some nanomaterials may be coated or have their surfaces modified by targeting homing antibodies or imaging contrast agents to achieve more desirable properties for treatment and diagnostics. If surface modification will be used, its effect on PT should be considered by researchers when designing their experiments.

PT also enhances nanomedicine in a variety of manners, further proving its combinatorial potential. PT has shown to induce vascular changes, which can alter the permeability and retention of NP accumulation in tissues, particularly in tumorous tissues. Additionally, a physician can use PT to more precisely control drug release or activation using nanomaterials, particularly in utilizing PCI and the use of light in these cases can minimize off-target release or activation, further enhancing the safety of therapeutics. Nanomedicine, therefore, benefits greatly from combination with PT, as each helps to compensate for the other's weaknesses.

### C. PT + immunotherapy

**FIG. 7.**
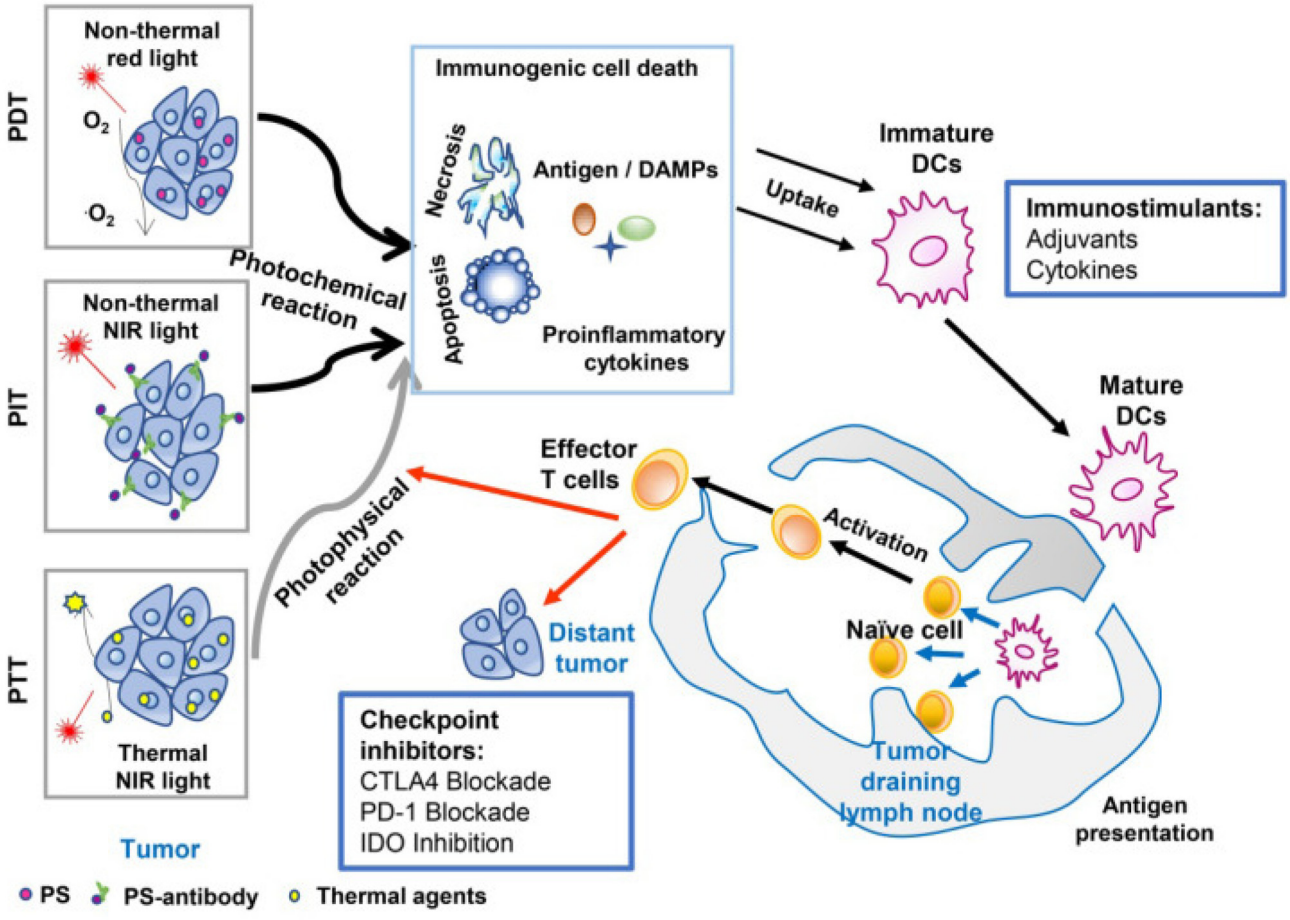
An overview of cancer treatment using the combination of phototherapy and immunotherapy. Agents absorbed energy from light to kill tumor cells, by photochemical reaction in the case of PDT (Photodynamic therapy) and PIT (Photoimmunotherapy), or photophysical reaction in the case of PTT (Photothermal therapy). Induced tumor cell death with the release of antigens, DAMPs, and proinflammatory cytokines, can provide *in situ* autologous cancer vaccines. Immunoadjuvants or cytokines can enhance the antigen capture and presentation by APCs, which will amplify the subsequent systemic immune response, resisting the residual tumor cells in the primary sites while allowing the host to establish a long-term defense against homologous cancer. Checkpoint inhibitors (antibodies against PD‐L1, antibodies against CTLA-4, or small molecule IDO inhibitors) can further improve the treatment efficacy by blocking the immunosuppressive receptors on the cell surface, restoring the cytotoxic function of tumor-specific T-cells. Reproduced with permission from Wang *et al.*, Theranostics **11**(5), 2218 (2021). Copyright 2021 authors, licensed under a Creative Commons Attribution (CC BY) license.

Combining immunotherapy and PT is a relatively new idea, originally conceptualized to address some of the problems each treatment faces respectively. Due to the large range of options for immunotherapy, researchers have many paths to take when designing experiments for these combinations. Several of these approaches can be seen in Fig. 7, treatments like stimulants, adjuvants, and cytokines can be combined with PDT and PTT to create robust antitumor responses.^116^ Efforts have been focused on increasing the specificity of immunotherapy and the potency of PT, especially on targets beyond the primary tumor. Table III presents the immunotherapy and PT combinations covered by this review.

**TABLE III.** Results of the combination of immunotherapies and PT. SLP—synthetic long peptide, Vac—vaccine, PDT—photodynamic therapy, IT-DC—intratumoral injection of dendritic cells, ACT—adoptive cell transfer, GC—glycated chitosan, PTT—photothermal therapy, NIR-PTT—near-infrared PTT, IM-NS—immunomodulator nanosystem, NP—nanoparticle, and PCI—photochemical internalization.

Tumor model species	Treatment	Major results	Reference
TC-1 tumors in mice	SLP Vac + PDT	Combination leads to systemic immune response	117
CT26 and B16 tumors in mice	IT-DC + PDT	Combination enhances tumor eradication, improved survival, and induced systemic immune response	118
EG.7-OVA in mice	ACT + PDT	Combination significantly delays tumor growth	119
DMBA-4 in rats	GC + PTT	Combination induces stronger immune response, eradicates primary and metastatic tumors, and provides long-lasting tumor resistance	23
B16 in mice	GC + NIR-PTT	Combination reduces tumor growth, increases survival, and induces long-term memory	120
MMTV-PyMT breast cancer in mice	GC + PTT	Combination enriches T cell population, increases tumor killing molecules, and upregulates genes associated with better survival in patients	121
Late-stage breast cancer in humans	GC + PTT	Objective response rate of 62.5%, clinical beneficial response rate of 75%, and no observed severe systemic adverse events	88
Panc02-H7 tumors in mice	GC + PTT	Combination leads to complete tumor regression, induces tumor-specific immune memory, demonstrates resistance to rechallenge, and enhances survival	122
MC38 tumors in mice	Neoantigen cancer Vac + PTT	Combination eradicates large primary tumors and induces a systemic response	123
CT26 tumors in mice	IM-NS + NIR-PTT	Combination inhibits tumor growth and induces systemic antitumor immune response	89
4T1 tumors in mice	Bioresponsive NP + PTT	Combination enhances antitumor immune response	124
*In vitro* and *in vivo* (mice) vaccination	Peptide antigen Vac + PCI	PCI efficiently delivers peptides and primes antigen-specific CD8+ T cell responses	125
EG.7-OVA cancer cells *in vitro*	Nanovaccines + PCI	Combination enhances reduction in tumor growth and immune response	126

#### 1. PDT + immunotherapy

Immunotherapy offers potent responses that, when combined with the specificity offered by PDT, create a powerful antitumor immune response. PDT can produce strong primary tumor ablation, which can then be combined with immunotherapy to induce the desired immune response. In 2016, Kleinovink *et al.* studied the effects of combining PDT with a therapeutic vaccine against a lung cancer model and a T-cell lymphoma model.^117^ Against the lung cancer model, Kleinovink *et al.* found that the combination of PDT with vaccination using synthetic long peptides containing tumor antigen epitopes significantly reduced tumor growth and increased survival, resulting in one third of the mice being cured.^117^ Additionally, the team found that this curative effect was not limited to the initial tumor, as it allowed the mice to resist rechallenge of the cancer model.^117^ Finally, the study showed that, while PDT alone is insufficient to destroy metastases, the combination of PDT and synthetic long peptides vaccination can eliminate secondary tumors in over 30% of mice treated, results that were mirrored in the lymphoma models.^117^

The combination of PDT and the introduction of immune cells to bolster the immune response is also a growing area of research for combination therapies. For example, Saji *et al.* investigated the post-irradiation injection of naïve dendritic cells against colorectal cancer and melanoma.^118^ They showed that the combination of PDT and the intratumoral injection of dendritic cells led to significantly improved survival in both tumor models as well as a systemic response in the mice, as evidenced by the regression of tumors at distant sites within the mice.^118^ More recently, Blaudszun *et al.* demonstrated utilizing PS-loaded T cells to combine adoptive cell transfer and PDT against a lymphoma model in mice.^119^ Following injection of the PS-loaded cytotoxic T lymphocytes (CTLs), the tumor was irradiated when the highest amount of PS-loaded CTLs was present in the tumor (which was determined by the team prior to treatment). The team showed that their combination therapy reduced tumor burden without compromising the overall health of the mice.^119^ Altogether, these results show that combining PDT with various immune therapies holds great potential for the complete eradication of cancer within patients, tackling both primary and secondary tumors while reducing off-target effects in patients.

#### 2. PTT + immunotherapy

**FIG. 8.**
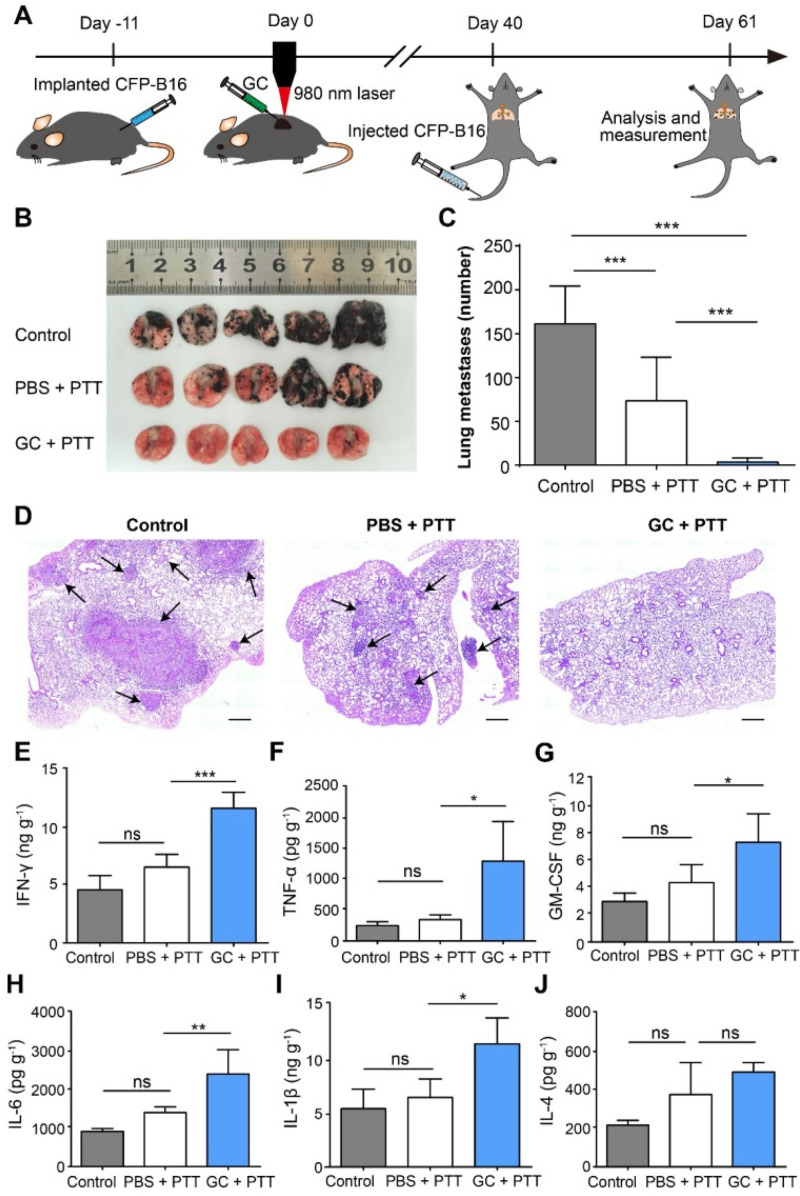
Inhibition of lung metastasis by LIT. (a) Schematics of the procedures and timeline of LIT in the inhibition of CFP-B16 tumor lung metastasis. (b) Tumor nodules in the lungs. Lungs collected from mice of different groups 21 days after 2 × 10^5^ CFP-B16 tumor cells were injected through tail vein. (c) Number of metastases in the lungs of different mice groups. Data are presented as mean ± SD (n = 9–12 mice, two independent experiments). (d) H&E staining of lung tissues of different mice groups. Scale bar: 500 *μ*m. (E)–(J) Cytokine levels (IFN-γ, IL-1 β, GM-CSF, IL-6, TNF-α, and IL-4) in the lungs collected from mice in different groups and were analyzed using ELISA. Data are presented as mean ± SD (n = 3–5 mice, two independent experiments). Statistical analysis was performed using the one-way ANOVA test followed by the Bonferroni post-test. ^*^ P < 0.05, ^**^ P < 0.01, ^***^ P < 0.001, ns: not significant. Reproduced with permission from Qi *et al.*, Theranostics **10**(4), 1814 (2020). Copyright 2020 authors, licensed under a Creative Commons Attribution (CC BY) license.

Like PDT, PTT is excellent in eliminating primary tumors but often falls short when trying to address metastases and provide long-term immunity. To this end, immunotherapy offers a way to enhance PTT's natural capabilities and make it more capable of targeting secondary tumors and providing a long-term benefit to patients. One popular method of boosting PTT involves the use of an immune stimulant/adjuvant to provide a noticeable boost to the immune system. Chen *et al.* showed combining PTT with the immunostimulant GC enhances the initial antitumor response in a murine DMBA-4 model and provides a lasting benefit to those who become tumor-free.^23^ Qi *et al.* found that PTT could be combined with GC to ablate the primary tumor and induce an immune response against an *in vivo* melanoma tumor model.^120^ Specifically, the team investigated how the treatment interacted with the immune system, showing that the combined treatment triggered a rapid response from neutrophils and led to long-term immune memory against recurrence and metastasis of the tumor model. As shown in Fig. 8, the combination of GC and PTT significantly reduced the number of lung metastases and increased the presence of anti-tumor cytokines, such as interferon gamma and tumor necrosis factor alpha.^120^

Hoover *et al.* showed that a combination of ablation (including PTT) and GC leads to the eradication of the primary tumor and drives a long-term immune response in a breast cancer tumor model.^121^ The group also reported that ablation alone, such as through PTT, could still induce an antitumor immune response, but that ablation should be supplemented with other treatments to achieve its full potential. With these results and further work by Chen, Nordquist, and other collaborators, PTT + GC eventually made its way to clinical trials for patients with late-stage breast cancer.^88^ Furthermore, Zhou *et al.* continued to study the effects of GC, focusing on pancreatic cancer in a 2018 study.^122^ In this study, the team found that the combination of PTT and GC led to significantly increased tumor regression and mouse survival.^122^ This benefit was not merely transient or local; the treatment provided mice with tumor specific systemic and long-term benefits, allowing them to resist rechallenge in the pancreatic model used, but not to a different tumor model (B16-F10).^122^

Stimulant/adjuvants are not the only method of boosting PTT performance, however. Nam *et al.* studied the use of a neoantigen cancer vaccine and PTT against both primary tumors and metastases.^123^ While each treatment could effectively handle smaller tumors, the team observed that each treatment struggled with larger primary tumors. Therefore, they hypothesized that combining the treatments may result in a more robust treatment of larger tumors, as each would be able to overcome the other's weaknesses. From their research, Nam *et al.* demonstrated that not only did the combination therapy help eliminate larger tumors, which each individual treatment could not, but the combination therapy also initiated a systemic response within the body, targeting distant metastases.^123^

Nanomedicine is often combined with PTT and immune therapies to provide a more vigorous treatment, such as the system developed and studied by Duan *et al.*^89^ In this study, Duan *et al.* found that their combined nanosystem, consisting of a PTA and an immunomodulator, and NIR-PTT treatment regimen increased the number of CTLs infiltrating the tumor, reduced tumor burden, and increased survival in mice against a colon carcinoma model.^89^ Similarly, Wang *et al.* developed a NP system consisting of a PS, ICG, and the Toll-like-receptor-7 agonist imiquimod into calcium carbonate NPs and tested this system in a 4T1 murine model.^124^ The NP platform decreased tumor growth rates and increased survival in mice, with the NP containing imiquimod resulting in significantly greater survival rates.^124^ Additionally, the team found that their system induced an immune response within the mice, including dendritic cell maturation and an increase in CD4+ and CD8+ T cell populations.^124^ PTT, due to its specificity and versatility, makes for a prime candidate in various synergistic therapies, especially regarding immunotherapies. Research into this crossover of fields continues to be important, as it may eventually lead to a more complete treatment for a wide variety of cancers.

#### 3. PCI + immunotherapy

The precise delivery of therapeutics is often a roadblock for immunotherapies, which is why PCI makes such an excellent option for the delivery mechanism. PCI allows physicians to dictate when a certain therapy is released using light, thereby limiting adverse side effects. Additionally, PCI combined with immunotherapy opens the doors for therapies, which may otherwise have difficulty in reaching their target. One example of this latter point can be seen in Haug *et al.*'s 2018 paper.^125^ In this paper, Haug *et al.* showed the efficacy of using PCI to deliver peptide antigens for cancer vaccination purpose. They found that their proposed mechanism significantly enhanced antigen-specific CTL responses both *in vitro* and *in vivo*, triggering responses that the antigen peptides alone previously could not induce due to poor immunogenicity.^125^

Cao *et al.* investigated the use of PCI to induce the self-assembly of nanovaccines against a lymphoma model.^126^ To accomplish this, the team developed a proto-nanovaccine consisting of an ICG derivative, ICG-sulfo-Osu, conjugated to ovalbumin (OVA), which serves as a model antigen. The team hypothesized that the ICG-OVA conjugate would self-assemble into a nanovaccine in an aqueous solution and that the release of this nanovaccine could be enhanced through PCI-induced endosomal and lysosomal escape into the cytosol for MHC-I antigen presentation.^126^ The team found that PCI significantly enhanced the cross-presentation of OVA and that the laser-ICG-OVA group exhibited significantly greater reduction in tumor volume than other groups.^126^ The team also observed an increased CD8+ T cell presence in the spleen and tumor, especially of CD8+ T cells associated with OVA, further demonstrating the enhanced immune response from the laser-ICG-OVA group over the non-laser group.^126^ These two studies, as well as many others, show the potential for PCI to be used to enhance the delivery of anticancer drugs to otherwise difficult to reach or previously unreachable targets.

#### 4. Insights on PT + immunotherapy

Immunotherapy and PT demonstrate excellent synergy together. In particular, immunotherapy aims to supplement PT by inducing a stronger immune response, targeting metastases that would be missed by the localized PT. Metastases are a recognized weakness of PT, therefore enhancing PT's ability to target them greatly increases the efficacy of the overall treatment. In this regard, immunotherapy can also help to clear any residual disease in the primary treatment area. Furthermore, phototherapy induces immunogenic cell death which can release tumor antigens and damage-associated molecular patterns, priming the immune system for immunotherapy and further targeting of metastases. PT can also favorably modulate the tumor microenvironment through the reduction of immunosuppressive cells and increasing the infiltration of cytotoxic T cells, making it more susceptible to immunotherapy. Finally, the synergy between immunotherapy and PT allows physicians to overcome tumor resistance mechanisms against monotherapy, a persistent issue in cancer treatments. This combined approach helps overcome common resistance mechanisms: PT boosts tumor immunogenicity and makes immunotherapy more effective, while immunotherapy targets metastatic spread beyond PT's reach.

### D. PT + immune checkpoint inhibitors

One of the key concerns in treating cancer is the possibility of the tumor mounting an anti-immune response. This is especially relevant when considering tumor escape mechanisms. A common mechanism deployed by cancer is the use of checkpoints. As covered earlier in this review, these ICs normally act as safety measures for our bodies, but their overexpression is exploited by certain cancers to prevent the immune system from properly targeting and eliminating the tumor. ICI, therefore, is an excellent measure against cancers utilizing these kinds of anti-immune strategies and is a common addition to several types of cancer therapy to bolster their effects. This is true for PT especially, as the combination of ICI and PT remains a productive area for study. Table IV introduces the ICI and PT combinations presented in this review.

**TABLE IV.** Results of the combination of immune checkpoint therapies and PT. 1MT—an immune checkpoint inhibitor, PDT—photodynamic therapy, CTLA-4—anti-cytotoxic-T lymphocyte associated protein 4, PD-L1—programmed death ligand 1, PD-1—programmed death protein 1 GNS—gold nanostars, PTT—photothermal therapy, NIR-PTT—near infrared PTT, SP—superparticle, and PCI—photochemical internalization.

Tumor model species	Treatment	Major results	Reference
CT26 tumors in mice	1MT + PDT	Combination strengthens antitumor response, particularly in inhibiting metastasis	128
MC38 tumors in mice	Anti-CTLA-4 and anti-PD-L1 + PDT	Combination prolongs survival and inhibits solid tumor growth	129
4T1 tumors in mice	Anti-PD-L1 + PDT	Combination significantly inhibits primary and metastasized tumor growth	130
4T1 tumors in mice	Anti-PD-1 + PDT	Combination significantly reduces growth of metastases and increases survival	131
MB49 tumors in mice	GNS + anti-PD-L1 + PTT	Combination reduces tumor size, increases survival, instills immunity, and enhances immune response	132
4T1 tumors in mice	Anti-CTLA-4 + NIR-PTT	Combination eradicates primary tumors, inhibits metastases, enhances maturation of DCs, and promotes production of anti-tumor cytokines	90
4T1 tumors in mice	SPs with anti-PD-L1 + NIR-PTT	Combination destroys primary tumor, triggers strong antitumor immune response, and inhibits metastases and distant tumors	91
4T1 tumors in mice	Anti-CTLA-4 + PTT	Combination decreases tumor burden, increases survival, and imparts resistance to tumor rechallenge	136
PD-L1+ and PD-L1- cancer cell lines *in vitro*	Anti-PD-L1 + PCI	Combination shows stronger cytotoxic effects in PD-L1+ cells and not in PD-L1- cells	24
CT26 and MC-38 tumors in mice	Anti-CTLA-4 + PCI or PDT	Combination with PCI shows enhanced anti-CTLA-4 efficacy compared to combination with PDT	137

#### 1. PDT + ICI

Patients often respond poorly to immunotherapies, including ICI, due to a lack of sufficient T cell response, tumor mutational burden, prevention of tumor killing by the TME, and other factors.^127^ Therefore, ICI is often combined with other therapies, such as PT or chemotherapy, to achieve maximal clinical success. PDT is one option for a combination partner, since it leads to tumor elimination and can prime an immune response, which can be advantageous for ICIs. Song *et al.* demonstrated the effects of combining PDT with an ICI in their 2018 paper on enhanced immunotherapy.^128^ In this study, an ICI known as 1MT was utilized, which blocks an immunosuppressive enzyme known for causing apoptosis of CTLs and differentiation of new regulatory T cells.^128^ While 1MT displayed some anticancer effects as a monotherapy, it showed increased efficiency in recruiting CTLs and an enhanced immune response when combined with PDT via the elimination of both primary and metastasized tumors in a colon carcinoma model.^128^

**FIG. 9.**
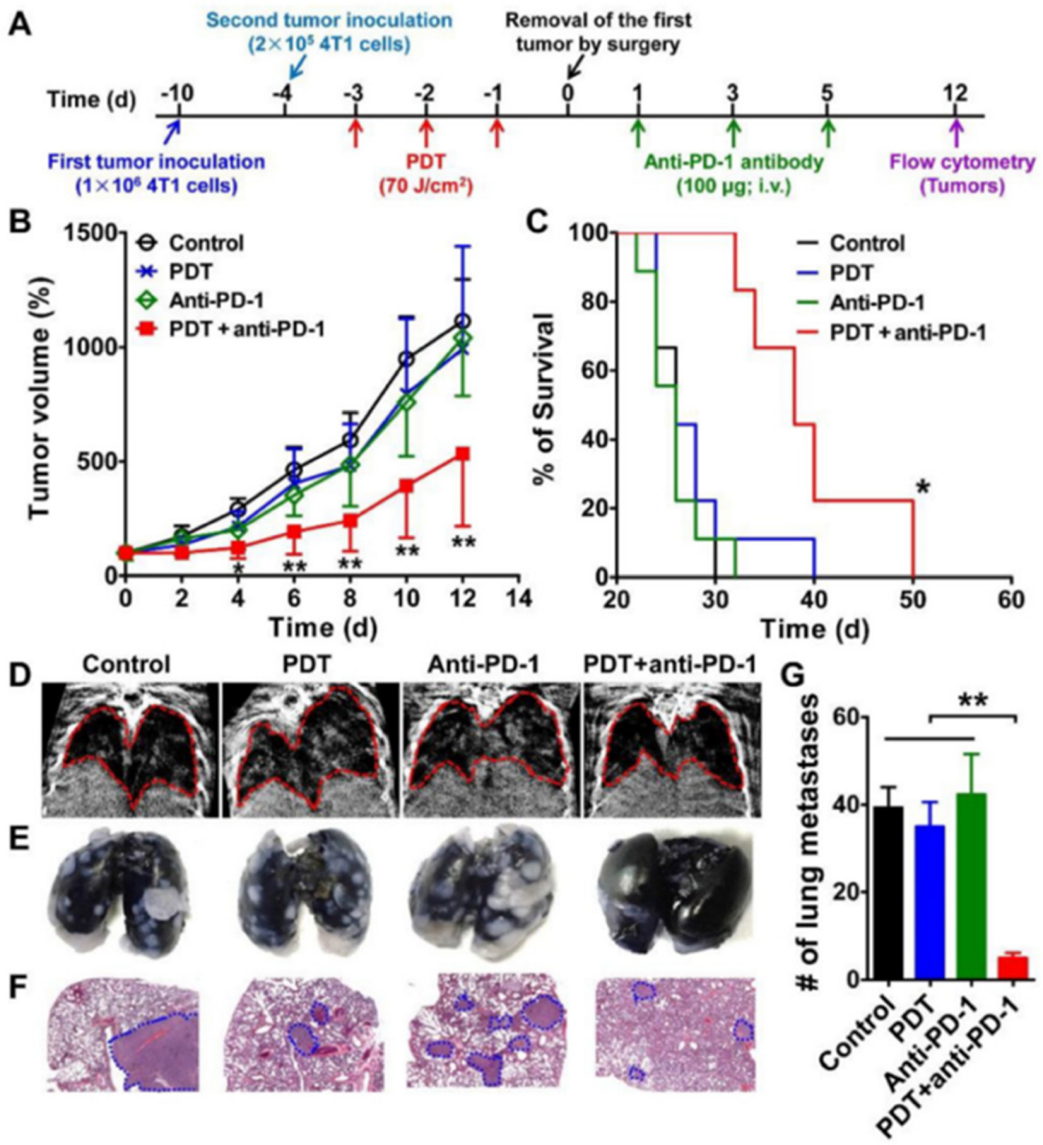
Inhibition of the growth and metastasis of the second tumor in mice treated with DSAB-HK PDT of the first tumor followed by PD-1 blockade. (a) Scheme of DSAB-HK PDT and PD-1 blockade combination therapy. (b) and (c) Growth curves the second 4T1 tumors in mice (b) and mouse survival curves (c) after different treatments of the first tumor and then removal the first tumor by surgery. (D)–(G) Representative images of *in vivo* CT scanning (d), India ink-filled lungs (e), H&E-stained lung sections (f), and the counted averages of tumor metastatic lesions in the lungs (g) from mice after the indicated treatments. Lungs in the CT images and tumor metastases in the H&E-stained lung slices are indicated by dashed circles. ^*^, P < 0.05; ^**^, P < 0.01. Reproduced with permission from Gao *et al.*, Theranostics **6**(5), 627 (2016). Copyright 2016 authors, licensed under a Creative Commons Attribution (CC BY) license.

Another major issue with both standard PDT and ICI is penetration into tumors. Because PDT relies on specific wavelengths of light, it may not penetrate larger tumors due to the physical constraints of light penetration. NIR light can alleviate this issue, so ICG was used to allow PDT to utilize this range of wavelengths. Hao *et al.* sought to understand how this NIR-PDT would interact with ICIs, specifically against CTLA-4 and PD-L1.^129^ The team found that the combination of NIR-PDT with both ICIs provided the strongest antitumor response, resulting in significantly stronger inhibition of tumor growth and the highest percentage of tumor-free survival rates.^129^ Looking to improve the outcomes of the combination of PDT and ICI, Wu *et al.* studied the effects of ICI timing on the antitumor efficiency of PDT.^130^ Their work in this study focused on 4T1 breast cancer, an immunologically “cold” and highly metastatic tumor model. They found that time plays a role on the efficacy of ICI following PDT therapy, noting that premature and belated ICI injection affects the outcome in different ways. Notably, premature ICI administration likely results in failure due to immature T cells, while T cell exhaustion likely causes belated ICI administration to fail.^130^ Gao *et al.* also studied 4T1 tumors, investigating the effects of an anti-PD-1 ICI in combination with PDT in a simulated metastasis model. Figure 9 illustrates their results, showing the combination of PDT and anti-PD-1 significantly reduced the growth rate of secondary tumors and enhanced the survival of mice.^131^

#### 2. PTT + ICI

PTT holds great potential as a combination partner due to its precise effect on tumors and non-crossover with other therapies. ICI has been a particularly intriguing potential partner for this therapy, given it can address weaknesses of PTT, such as the lack of systemic effects. Combined with other therapies, like nanomaterials, PTT + ICI has garnered further scientific interest in recent years. For example, Liu *et al.* developed a gold nanostar to use as a PTA for mild PTT, where the temperature remains within a hyperthermic range (less than 43 °C).^132^ This treatment reduced undesired tissue damage from high PTT, where the laser causes thermal ablation of the tumor, while still inducing the desired effects of improved drug delivery to the tumor, improved therapy sensitivity, and systemic immune responses.^133–135^ In their study using a bladder cancer model, Lie *et al.* demonstrated that their gold nanostar-PTT combined with an ICI for PD-L1 provided significant tumor growth inhibition and superior long-term tumor-free survival.^132^

**FIG. 10.**
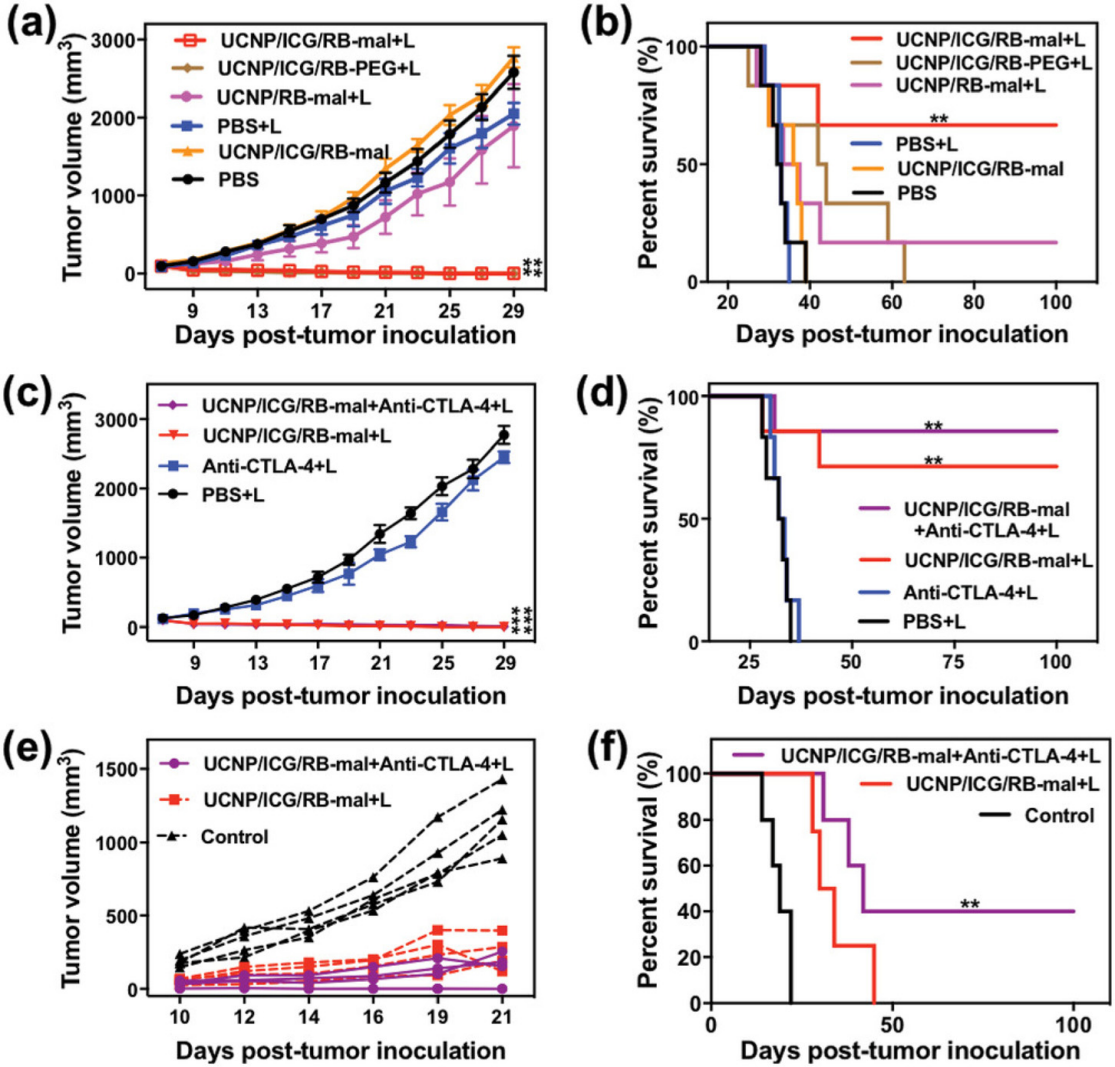
In vivo antitumor effect of UCNP/ICG/RB-mal. (a) Average tumor-growth curves of different treatment groups of mice with orthotopic 4T1 tumors (*n* = 6, ^**^P < 0.01 vs PBS group). (b) Survival curves of different treatment groups of mice bearing orthotopic 4T1 tumors (*n* = 6, ^**^P < 0.01 vs PBS group). (c) Average tumor-growth curves of different groups of treated mice with or without anti-CTLA-4 (*n* = 6, ^***^P < 0.001 vs PBS group). (d) Survival curves of different groups of mice-bearing orthotopic 4T1 tumors in (c) (*n* = 6, ^**^P < 0.01 vs PBS group). (e) Growth curves of tumors in individual mice successfully treated mice by phototherapy after tumor cell rechallenge. (f) Survival rates of mice in (e). (*n* = 4, ^**^P < 0.01 vs control group.) Data are expressed as mean ± SD. Reproduced with permission from Wang *et al.*, Adv. Sci. **6**(10), 1802157 (2019). Copyright 2019 authors, licensed under a Creative Commons Attribution (CC BY) license.

Other research teams have also investigated the use of nanomaterials in combination with PTT and ICI. In 2014, Wang *et al.* studied the use of SWNT-mediated PTT in combination with an anti-CTLA-4 ICI.^90^ In their *in vitro* studies, the team found that their SWNTs enhanced the maturation of dendritic cells, including against other common PTAs like ICG, gold nanorods, and GO; these results were also confirmed in their *in vivo* model.^90^ Additionally, they studied the effects of their SWNTs against metastasis, using a 4T1 tumor model in mice. While their SWNTs combined with PTT failed to fully inhibit the growth of a simulated metastasis, the team found that the addition of anti-CTLA-4 enhanced their treatment leading to significant inhibition of the secondary tumor and increased survival in their murine model.^90^ Similarly, Ge *et al.* constructed what they call a “superparticle” that can be loaded with nanodrugs and combined with anti-PD-L1 and NIR-PTT to create potent antitumor effects.^91^ These superparticles are unique in that they are partially ferrous, making them magnetic. As a result, the superparticles could be directed to destroy tumors when treated with NIR-PTT, and the addition of anti-PD-L1 results in the eradication of primary tumors, the inhibition of distant tumors, and the prevention metastasis to the lung or liver in a 4T1 cancer model.^91^ Another study, conducted by Wang *et al.*, investigated a NP system they developed in a murine 4T1 tumor model. This system consisted of upconversion NPs, which are frequently used in NIR-PDT and NIR-PTT, capped with ICG and a lipid molecule known as DSPE-PEG-mal and loaded with rose bengal, which acted as a PS.^136^ The team then combined this system with NIR-laser irradiation and anti-CTLA-4. Altogether, the NP-ICI-laser therapy improved animal survival and reduced tumor burden, as shown in Fig. 10.^136^ Additionally, Figs. 10(e) and 10(f) demonstrate the finding that combination therapy imparted lasting resistance to tumor rechallenge.^136^ Many pre-clinical and several clinical trials are currently investigating PTT + ICI combination therapy, given its potential as a powerful anticancer therapy and the promising results found in the above studies.

#### 3. PCI + ICI

One of PCI's greatest strengths is the ability to precisely control drug release via laser irradiation, leading to a highly efficient drug delivery mechanism. Therefore, ICI becomes a powerful tool when combined with PCI, as ICIs can be used as the drug to be delivered or the checkpoints themselves used as targets for delivery. This second approach is described in Wong and Selbo's 2021 paper titled “Light-controlled elimination of PD-L1+ cells.”^24^ In this study, Wong and Selbo precisely delivered an immunotoxin to triple negative breast cancer cells *in vitro* with PD-L1 as a target for cellular uptake. Because PD-L1 is taken up by endocytosis, the investigators developed a PD-L1-targeting immunotoxin, anti-PD-L1-saporin, and administered it to cells. From there, they irradiated the cells at different time points and measured the cytotoxic effects of the toxin. The investigators reported that their delivery mechanism is time- and light-dependent, with cellular viability decreasing significantly upon irradiation after an 18-h incubation.^24^ These results indicate the cytosolic release of drugs entrapped in endo/lysosomal vesicles by PCI. The investigators also tested a non-PD-L1-targeting variant of the immunotoxin, showing that it did not result in a significant cellular death upon irradiation. Furthermore, Wong and Selbo analyzed how the concentration of the two toxins affected cell viability. They found that only the anti-PD-L1 toxin showed increased cellular death at higher concentrations,^24^ suggesting that the targeting with PD-L1 helped increase cellular uptake of the toxin. They also demonstrated that PCI assists in delivering the toxin to cells and disruption of the endosomes via irradiation, as the irradiated groups saw a greater decrease in viability compared to the non-irradiated groups.^24^ Since this method focuses on using PD-L1 as a target, it can theoretically function in a variety of PD-L1-expressing cancer types.

PCI may also be combined with ICI to augment the overall therapeutic effect. Based on this approach, Longva *et al.* designed an experiment to test the effects of PCI enhancement of an anti-CTLA-4 therapy.^137^ The team used two different colon cancer cell lines in a murine model, CT26.WT and MC-38, to study the effects of PCI on the efficacy of anti-CTLA-4. Specifically, they used a vascular targeting toxic compound, which would be delivered intravenously and irradiated by a diode laser 6 h after injection. This compound consists of a toxin, gelonin, which is a type 1 ribosome-inactivating protein, that is linked to vascular endothelial growth factor 121 to form VEGF121/rGel.^138^ Weyergang *et al.* showed that PCI enhances the release of this toxin from endocytic vesicles into the cytosol and the toxicity of VEGF121/rGel.^139^ Building on this previous work, Longva *et al.* investigated how PCI-VEGF121/rGel affected the therapeutic benefits of an ICI, such as anti-CTLA-4. In both cell lines tested, the team observed that the combination of their PCI treatment and anti-CTLA-4 significantly reduced tumor burden and increased tumor clearance in mice.^137^ These results indicate that PCI holds great promise as an ICI enhancer. Additionally, PCI may be used as part of a delivery strategy for ICIs, though this is an area which should be more closely investigated.

#### 4. Insights on PT + ICIs

ICIs demonstrate exceptional synergy with PT. By combining these two types of therapy, clinicians can provide localized, specific anti-tumor effects via PT and systemic effects through ICIs, resulting in significantly enhanced efficacy and a broader treatment plan for patients. Specifically, ICIs target a common mechanism for tumor escape, immune checkpoints, allowing for a systemic response to an otherwise localized one with PT alone. PT can enhance ICI treatment by turning an otherwise immunologically cold tumor into an immunologically hot one, allowing ICI therapy to affect a broader range of cancer types. Furthermore, like other immunotherapies, ICI therapy is enhanced due to the release of tumor antigens and damage-associated molecular patterns via PT-induced immunogenic cell death, though ICIs have an advantage over other types of immunotherapies by leveraging the power of the patient's natural immune system and enhancing its action rather than merely priming the immune system. Finally, ICIs can be more precisely tailored to specific patients by analyzing tumor samples to determine the types of immune checkpoints the tumor is overexpressing and directly combatting the tumor with a specific ICI, reducing the off-target effects of the therapy. Therefore, it is evident that a careful combination of PT and ICIs can lead to improved patient outcomes. PT enhances ICI therapy by increasing immune cell infiltration while ICIs combat common tumor escape mechanisms.

## V. PHOTONICS IN CANCER THERANOSTICS

### A. Introduction to photonics-driven diagnostics

In addition to therapeutic applications of light, physicians can utilize photonic techniques to image and detect cancers more efficiently and precisely. When cancers are detected and treated at earlier stages, the chances of survival increase across numerous cancer types.^140^ Therefore, it is imperative that new and robust cancer detection methods are developed. These developments can arise from advancements in photonics, as photonics offers precise and tunable instruments and techniques which can greatly enhance diagnostics. For example, optical biosensors measure the absorbance, reflectance, or fluorescence of biological materials of interest^141^ while techniques such as Raman spectroscopy utilize energy shifts in scattered photons to identify the chemical structures of analyzed materials.^142^

Photonics imaging techniques have a long history, dating back to the sixteenth century with the creation of the first microscope by Zaccharias Janssen and Hans Janssen and the first observations of microorganisms using such technologies by Antoni van Leeuwenhoek in the seventeenth and eighteenth centuries.^143^ Since then, important advancements in the field have included the electron microscope in 1931 by Knoll and Ruska, improvements in contrast for thin objects during the early 1930s by Linnik (1931) and Zernike (1934), full color imaging in 1943 by Brumberg, and time-lapse filming by the Zeiss factory in 1943.^143^ Fluorescence imaging also made great strides during the late 19th and 20th centuries, with innovations in fluorescent dyes, microscopes, and staining techniques as well as the discovery of immunofluorescence by Coons and Kaplan.

Modern photonics research has been heavily focused on improving aspects of imaging such as range of analytes, specificity, and efficiency in imaging to increase patient outcomes in a variety of cases. Chatzipetrou *et al.* developed a photonic biosensor which could be used to monitor up to six different cancer biomarkers, such as periostin and transforming growth factor beta-induced protein. These two markers are commonly overexpressed by cancer stem cells and found circulating in the blood, meaning their detection can be an asset in identifying cancer patients in earlier stages of their disease.^144^ SPR utilizes thin metal sheets covered with capturing agents, like antibodies, to measure whether binding has occurred. When these capturing agents bind to their target, the refractive index of the sheet changes, which researchers can quantify to measure binding events.^145^ In a 2021 study, Yasli developed a photonic biosensor to detect cancer in its early stages which utilizes SPR. Combined with a special optical fiber consisting of periodic air holes against a silica background, the device demonstrated high specificity in identifying six different types of cancer cells, indicating promising results for further research into this methodology.^146^

In addition to biosensors, techniques involving photonics concepts, such as refractive index, are useful in detecting cancer in patients. For example, cancerous cervical cells possess a different refractive index than their healthy counterparts.^147^ Taking advantage of this difference, Kruczkowski *et al.* proposed a method of measuring the refractive index of cervical cells and using machine learning to rapidly and accurately identify cancerous cells. The team found that a Naïve Bayes model performed best, with an accuracy and precision of 92% and 93%, respectively, on the validation dataset along with the fastest training and prediction times among all models tested.^147^ As machine learning improves, this method will become even more accurate and precise, and one day, it might be applied to other cancer types with a similar difference in refractive index between healthy and cancerous cells to better assist physicians in diagnosing deadly cancers sooner.

In addition to biosensors and SPR, several other imaging modalities have grown in interest, leading to exciting research throughout the photonics world. Fluorescence microscopy takes advantage of the natural fluorescence of certain materials, allowing for highly specific images of multiple fluorescent tags in biological samples. While it has limitations, recent advancements in areas, such as super-resolution, have continued to drive research in this field. Another method is known as multi-photon microscopy, which takes advantage of short pulses of light to produce high contrast and deep penetration images of biological materials. However, because this technique is expensive, requires complex equipment, and has a slow acquisition time, it is not an ideal method of imaging. A more frequently used technique is known as optical coherence tomography (OCT) which has garnered great attention in photonics. Using a split beam of light and interference patterns, OCT allows researchers to non-invasively image tissues and samples in real-time with high resolution.

### B. Current trends in image-guided phototherapy

Photonics research does not end with diagnosis or treatment alone, the fusion of these two areas forming the field of theranostics. Theranostics seeks to combine various components of photonics-based research to simultaneously identify, monitor, and treat diseases like cancer. To this end, researchers have been focused on creating new modalities for the efficient combination of diagnostic and therapeutic work, especially in the field of image-guided phototherapy. Other areas discussed in this review play a role in this field of research, especially nanomaterials. Nanotechnology allows for a great range of customization within theranostic approaches, providing scientists with the capability to more effectively combat cancer in a wide variety of patients.

**FIG. 11.**
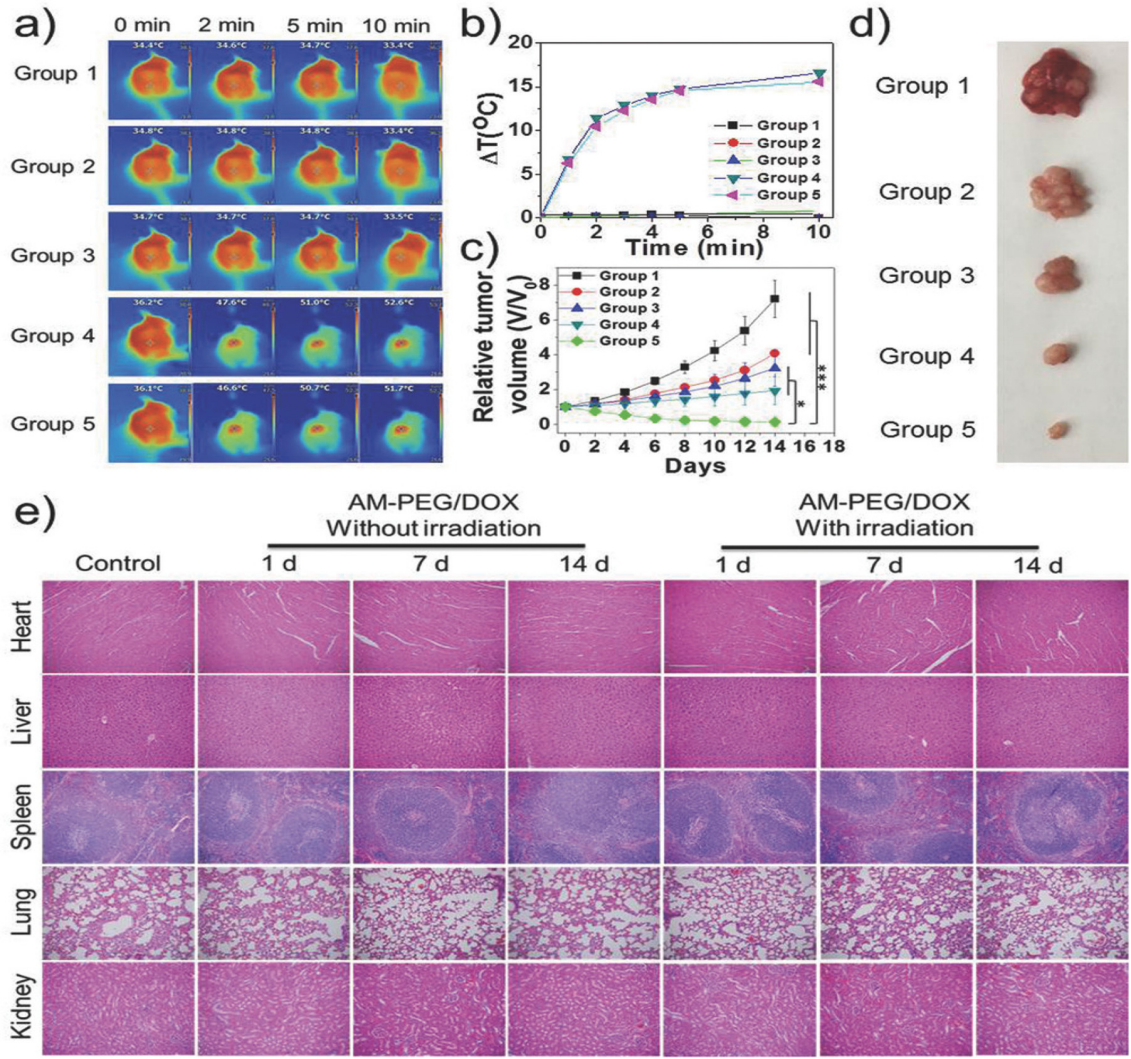
In vivo combined cancer therapeutic effect of AM‐based NSs. Group 1: saline; group 2: DOX; group 3: AM‐PEG/DOX NSs; group 4: AM‐PEG NSs + NIR; and group 5: AM‐PEG/DOX NSs + NIR. NIR laser irradiation was performed in Groups 4 and 5 for 10 min (0.8 W cm^−2^, 808 nm) after 12 h of i.v. injection ([AM] = 6 mg kg^−1^,[DOX] = 6 mg kg^−1^). (a) Infrared thermographic maps and (b) time‐dependent temperature changes in the MCF‐7‐tumor‐bearing nude mice after different treatments. (c) Growth curves of MCF‐7‐tumor‐bearing nude mice after different treatments (^*^P < 0.05, ^***^P < 0.001). (d) Digital photo of representative tumors in different groups after 14 D treatment. (e) H&E‐stained histological images of tissue sections from major organs (heart, liver, spleen, lung, and kidney) at 1, 7, and 14 D post‐injection with different treatments. Reproduced with permission from Tao *et al.*, Adv. Mater. **30**(38), 1802061 (2018). Copyright 2018 authors, licensed under a Creative Commons Attribution (CC BY) license.

Nanomaterials designed for theranostic purpose should possess biocompatibility, efficient tumor accumulation (especially deep tumor penetration), and improved therapeutic effects. One such nanomaterial is a 2D material derived from antimony known as antimonene (AM). In a 2018 paper, Tao *et al.* describes their testing of AM NSs in both *in vitro* and *in vivo* models. During their *in vitro* experiments, the team found that the AM NSs were biocompatible and provided laser-induced deep-tumor delivery of a chemotherapy drug of interest, doxorubicin (DOX).^148^ Tao *et al.* then investigated the therapeutic effects of their AM-DOX combination alongside laser irradiation. Following promising results from this experiment, the researchers used a mouse breast cancer model to evaluate the theranostic potential of their platform. The team first demonstrated the potential for *in vivo* fluorescence imaging, demonstrating stronger photoacoustic signals compared to black phosphorus nanomaterials. Furthermore, as shown in Fig. 11, the AM-DOX combination can simultaneously provide infrared imaging and therapy with the laser–nanosystem combination, resulting in a lowered tumor burden.^148^

Zhang *et al.* also created NSs to investigate their imaging and therapeutic effects in a 2022 study. Unlike Tao *et al.*, Zhang *et al.* used a special form of oxygen deficient tungsten oxide as the base of their NSs, hypothesizing it would possess favorable photothermal effects in the NIR-II window.^149^ The team showed that their NSs possessed strong NIR-II absorbance, which translated to a significant photothermal effect, and compatibility with multimodal *in vivo* imaging.^149^ Before Zhang *et al.*, Wang *et al.* investigated the NIR-II window in a 2019 study. Unlike the previous two example, Wang *et al.* developed a NP from titanium nitride and found the nanomaterial offered high photothermal-conversion efficiency and favorable biocompatibility.^150^ The titanium nitride NPs allowed the team to eradicate a murine breast cancer model while also serving as an efficient contrast agent for photoacoustic imaging, demonstrating a viable option for theranostic work.^150^ Table V summarizes the results of this section, highlighting the range of possibilities for theranostic work.

**TABLE V.** Results of the combination of cancer therapies and diagnostics. SPR—surface plasmon resonance, PCF—photonic crystal fiber, RI—refractive index, ML—machine learning, AM-NS—antimonene nanosheets, NIR-PTT—near-infrared photothermal therapy, DOX—doxorubicin, TO-NS—tungsten oxide nanosheets, and TiN-NP—titanium nitride nanoparticles.

Tumor model species	Treatment/diagnostic technique	Major results	Reference
Breast cancer patient serum	Photonic biosensor	Sensor detects two different biomarkers associated with cancer model	144
Basal, HeLa, Jurkat, PC12, MDA-MB-231, and MCF-7 cells *in vitro*	SPR-PCF biosensor	Sensor differentiates all six cancerous cell lines from healthy cells with high sensitivity	146
Cervical cancer cells	Optoelectronic sensor measuring RI + ML	Combination achieves 92% accuracy and 93% precision in identifying cancerous cells	147
MCF-7 *in vitro* and *in vivo*	AM-NS + NIR-PTT + DOX	Combination provides deep tumor delivery of DOX, therapeutic effects, and robust imaging capabilities	148
4 T1 *in vitro* and *in vivo*	TO-NS + NIR-PTT + DOX	Combination provides multimodal imaging capabilities and tumor eradication	149
4 T1 *in vitro* and *in vivo*	TiN-NP + NIR-PTT	Combination eradicates tumors while nanoparticle acts as contrast agent	150

PT possesses several qualities which make it ideal for enhancing theranostics, making it an excellent foundation for this combination. The primary quality is that many agents used in PT, such as PSs and PTAs, inherently possess or can be modified to possess properties that permit them to be used as imaging agents. For example, many PSs and PTAs are fluorescent, allowing them to effectively perform as theranostic agents since the same molecule used in the therapy can be imaged. However, this idea can also apply to certain nanomaterials, so why focus on PT? PT uses light as its trigger for its therapeutic effects, and light is a common signal generation method for imaging. Therefore, PT can be activated while imaging occurs. There are other advantages to using PT in theranostic applications. For example, by imaging while treating, physicians and researchers can gain insight into the mechanisms of treatments, as simultaneous imaging and treatment will provide measurable markers through specific cellular changes. Researchers can use theranostic techniques to measure the accumulation and distribution of PT agents before and during treatment, monitor the biochemical effects of PT (such as ROS generation), and quantify the downstream cellular responses induced by PT through the observation of cellular death markers. This ability of PT-based platforms to combine imaging and treatment using light activation not only allows for precise monitoring and control but can also streamline the clinical workflow, potentially shortening the crucial time between cancer detection and intervention.

## VI. CONCLUSION AND FUTURE DIRECTIONS

Cancer is a devastating disease, significantly impacting the lives of patients and those around them. Over the past several decades, remarkable progress has been made in the development of cancer diagnostics and therapies, from surgery, chemotherapy, and light microscopy to nanomedicine, immunotherapy, PT, and advanced imaging techniques like SPR and OCT. Individual therapies have their own unique advantages and disadvantages against cancer, and their differences should be further investigated to design the most effective treatment for each patient. Particularly, PT provides specific and potent local responses, is minimally invasive, and is an excellent choice for combination therapy. However, it possesses limitations as a monotherapy due to a lack of systemic and long-lasting effects against cancer. Meanwhile, other therapies like immunotherapy and nanomaterials can provide powerful, long-lasting protection against cancer but often lack specificity and tumor penetration, leading to undesired side effects and poor clinical outcomes.

Identifying the need for a new approach, researchers have begun investigating the combination of various therapies, especially that of PT, nanomedicine, and immunotherapy with each other, and diagnostic technologies. While many proposed therapies still lack proper clinical trial data, pre-clinical studies have shown promising results. Therefore, it is imperative that researchers continue to study the various combinations of these therapies and diagnostic techniques. A focus should be on optimizing current procedures and parameters and identifying synergies so the most efficient treatments can begin clinical trials. However, it should be kept in mind per Hoover *et al.*, “Each TME is unique… It is for this reason that a one-size-fits-all cancer approach has had little success.”^127^ No single treatment, nor a specific combination, will work for every patient, which should be considered when designing experiments and treatment plans. Therefore, it is important to better understand how each treatment interacts with the immune system, both in general and on a per-patient basis. This includes taking advantage of cutting-edge diagnostic tools and techniques to more accurately identify and monitor cancers in real-time.

When developing novel treatments, researchers should consider several critical questions. These include the optimal dosage and timing of combination therapies, potential side effect profiles, and patient conditions. The synergy between different components of a regimen necessitates optimization studies to determine the most effective dosing and scheduling. Furthermore, the efficacy of certain therapies, such as combinations using ICIs, is significantly influenced by tumor characteristics, a factor that scientists must account for in their investigations. Finally, other treatments, such as chemotherapy, immunotherapy, and surgery, may be excellent candidates for combination with phototherapy, offering a wide array of options for future research.

As shown in Sec. IV of this paper, combination therapies hold significant promise in achieving this goal, as their customizability gives them a unique advantage over monotherapies and offers greater effectiveness against a multitude of cases. When combined with diagnostic methods, the synergy between photonics-based PTs and other therapies, particularly immunotherapies, becomes apparent. Together, photonics-focused cancer theranostics holds great potential to provide a systemic solution to cancer, a disease initiated from our own human cells and with an immunological root.

## VII. METHODS

### A. Literature search strategy

A comprehensive literature search was conducted using databases such as PubMed and Google Scholar. Keywords, including “photothermal therapy,” “photodynamic therapy,” “nanomaterials photothermal therapy,” and “immune therapy phototherapy,” were used. Articles published in peer-reviewed journals were considered. Additional sources were identified through cross-referencing relevant articles.

### B. Inclusion and exclusion criteria

Studies were included if they discussed nanomaterials, immune therapies, and phototherapies, combinations of non-phototherapies and phototherapies in preclinical or clinical settings, and provided experimental or review-based insights. Articles were excluded if they lacked relevance to phototherapy, relevant non-phototherapies, or the combination of these therapies, were not in English, or did not provide sufficient methodological details.

### C. Quality assessment

For primary research articles, study design, sample size, reproducibility, and statistical rigor were evaluated. Reviews were assessed for comprehensiveness and citation relevance. Bias was minimized by independently verifying critical data points across multiple sources.

## Data Availability

Data sharing is not applicable to this article as no new data were created or analyzed in this study.
